# Future cost-effectiveness and equity of the NHS Health Check cardiovascular disease prevention programme: Microsimulation modelling using data from Liverpool, UK

**DOI:** 10.1371/journal.pmed.1002573

**Published:** 2018-05-29

**Authors:** Chris Kypridemos, Brendan Collins, Philip McHale, Helen Bromley, Paula Parvulescu, Simon Capewell, Martin O’Flaherty

**Affiliations:** 1 Department of Public Health and Policy, University of Liverpool, Liverpool, United Kingdom; 2 Public Health Department, Liverpool City Council, Liverpool, United Kingdom; Edinburgh University, UNITED KINGDOM

## Abstract

**Background:**

Aiming to contribute to prevention of cardiovascular disease (CVD), the National Health Service (NHS) Health Check programme has been implemented across England since 2009. The programme involves cardiovascular risk stratification—at 5-year intervals—of all adults between the ages of 40 and 74 years, excluding any with preexisting vascular conditions (including CVD, diabetes mellitus, and hypertension, among others), and offers treatment to those at high risk. However, the cost-effectiveness and equity of population CVD screening is contested. This study aimed to determine whether the NHS Health Check programme is cost-effective and equitable in a city with high levels of deprivation and CVD.

**Methods and findings:**

IMPACT_NCD_ is a dynamic stochastic microsimulation policy model, calibrated to Liverpool demographics, risk factor exposure, and CVD epidemiology. Using local and national data, as well as drawing on health and social care disease costs and health-state utilities, we modelled 5 scenarios from 2017 to 2040:

1. Scenario (A): continuing current implementation of NHS Health Check;
2. Scenario (B): implementation ‘targeted’ toward areas in the most deprived quintile with increased coverage and uptake;
3. Scenario (C): ‘optimal’ implementation assuming optimal coverage, uptake, treatment, and lifestyle change;
4. Scenario (D): scenario A combined with structural population-wide interventions targeting unhealthy diet and smoking;
5. Scenario (E): scenario B combined with the structural interventions as above.

We compared all scenarios with a counterfactual of no-NHS Health Check.

Compared with no-NHS Health Check, the model estimated cumulative incremental cost-effectiveness ratio (ICER) (discounted £/quality-adjusted life year [QALY]) to be 11,000 (95% uncertainty interval [UI] −270,000 to 320,000) for scenario A, 1,500 (−91,000 to 100,000) for scenario B, −2,400 (−6,500 to 5,700) for scenario C, −5,100 (−7,400 to −3,200) for scenario D, and −5,000 (−7,400 to −3,100) for scenario E. Overall, scenario A is unlikely to become cost-effective or equitable, and scenario B is likely to become cost-effective by 2040 and equitable by 2039. Scenario C is likely to become cost-effective by 2030 and cost-saving by 2040. Scenarios D and E are likely to be cost-saving by 2021 and 2023, respectively, and equitable by 2025. The main limitation of the analysis is that we explicitly modelled CVD and diabetes mellitus only.

**Conclusions:**

According to our analysis of the situation in Liverpool, current NHS Health Check implementation appears neither equitable nor cost-effective. Optimal implementation is likely to be cost-saving but not equitable, while targeted implementation is likely to be both. Adding structural policies targeting cardiovascular risk factors could substantially improve equity and generate cost savings.

## Abstract 2

In a modeling study, Chris Kypridemos and colleagues assess the NHS Health Checks program for cardiovascular risk screening in Liverpool, UK.

## Author summary

**Why was this study done?:**

- Previous evidence for population cardiovascular screening (e.g., National Health Service [NHS] Health Check in England) has failed to conclusively provide answers around effectiveness, cost-effectiveness, and equity.
- A recent systematic review found some evidence of cost-effectiveness, but some of the studies included were methodologically flawed.

**What did the researchers do and find?:**

- The researchers simulated the life courses of synthetic individuals under different scenarios, within a close-to-reality synthetic population of Liverpool.
- This study provides detailed evidence on effectiveness, cost-effectiveness, and equity of health checks while also uniquely providing estimations of the speed at which these programmes will reach these thresholds.
- The results show that, while different implementation structures for health checks may improve cost-effectiveness and equity, these approaches are dominated by the addition of population-wide, structural interventions to improve diet and reduce smoking (e.g., mandatory salt reformulation of processed foods or increased taxation on tobacco products).
- Liverpool is a city with a high concentration of cardiovascular risk factors and high burden of cardiovascular disease (CVD), and therefore the likelihood of programme effectiveness increases.

**What do these findings mean?:**

- The findings highlight the importance of a carefully considered approach to the implementation of health checks while also considering other approaches for CVD prevention.
- The addition of structural interventions will lead to primary CVD prevention becoming equitable and cost-effective earlier.

## Introduction

Cardiovascular disease (CVD) is a leading cause of morbidity and mortality globally [1]. In 2015, almost 500,000 deaths across the United Kingdom were attributed to CVD, more than any other cause of death [2]. As part of the prevention of CVD, the National Health Service (NHS) Health Check programme was implemented across England in 2009. Local-authority commissioning of NHS Health Check has been a statutory requirement since 2013 as part of the Health and Social Care Act [3]. The programme involves CVD risk stratification—at 5-year intervals—of all adults between the ages of 40 and 74 years, excluding any with known preexisting vascular conditions (including CVD, diabetes mellitus, and hypertension, among others) [4]. Those identified as high-risk are offered appropriate treatment, including pharmacological and behavioural interventions. There are limited data available on the national programme. There is little consistency in commissioning arrangements; however, most areas use local General Practices (GPs).

There is conflicting evidence regarding the effectiveness—and cost-effectiveness—of health checks. In the UK, cost-effectiveness for public health interventions is often compared to a threshold of £20,000 per quality-adjusted life year (QALY) gained [5]. The programme was modelled by the UK Department of Health in 2008 [6], suggesting that health checks were cost-effective. However, concern was raised that assumptions about the effectiveness of lifestyle interventions might have been overestimated. A more recent study looking only at the weight-loss impact of NHS Health Check found them cost-effective, through attributing the weight-loss effect exclusively to health checks [7]. A Cochrane review found that, while health check programmes increased diagnosis, they had no significant effect on survival; however, the majority of studies in the review were from before 1980, prior to the introduction of many pharmacological interventions (such as statins for hypercholesterolaemia) [8]. A systematic review of economic evaluations for health checks reported that some modelling and observational studies found programmes to be cost-effective. However, some of these were methodologically flawed (i.e., pre–post designs without a control group) and were based on assumptions regarding uptake and treatment effectiveness that are not supported by empirical evidence [9]. A recent study has suggested that targeting health checks to those most at risk might increase cost-effectiveness [10].

There are also concerns about the potential effect of health checks on health inequalities. An analysis of the first 4 years of the NHS Health Check programme suggested low, but improving, overall uptake and higher uptake in the most deprived communities. However, this did not incorporate a complete 5-year cycle; therefore, it may reflect that, in some areas, the invitation strategy prioritised people with lower socioeconomic circumstances [11]. A rapid systematic review for the Expert Scientific and Clinical Advisory Panel identified that there was wide variability in uptake across England, and the evidence regarding differential uptake by sex, ethnicity, or deprivation was inconclusive [12]. Public Health England (PHE) recently suggested that the programme could potentially be successful in reducing inequalities, but this would require high and equitable uptake in high-risk groups [13]. Local authorities have the flexibility to focus more resources on high-risk communities, making a targeted approach on top of universal coverage possible through an approach of proportionate universalism [14]. A microsimulation study suggests that this could increase the equity of the intervention [15]. Yet there is continuing concern that interventions that target high-risk individuals such as the Health Check programme might exacerbate health inequalities given that they have the potential to favour populations with higher levels of resources [16,17].

On the other hand, population-wide policy approaches to reducing CVD can be effective, cost-effective, and improve health inequalities [16]. These approaches typically focus on tobacco and alcohol control as well as dietary interventions, such as mandatory salt reformulation of processed food or a sugar-sweetened beverage (SSB) levy [18–23]. Therefore, the optimal combination of individual and population-level strategies to reduce the unequal burden of CVD is still not well-defined.

The current study focusses on Liverpool, a city in northwest England with high levels of socioeconomic deprivation. Liverpool is ranked the fourth most deprived local authority in England when considering the proportion of neighbourhoods in the most deprived quintile [24]. Furthermore, there are high levels of inequality within the city, with notable clustering of CVD risk factors that might favour targeted, high-risk approaches to prevention. Health checks were implemented in Liverpool in 2010, but this remains suboptimal; an audit of the programme in 2015 and 2016 found large variations in practice [25]. Barely two-thirds of eligible residents were invited, of which less than one-third completed a health check. These figures are below the national average of about 86% and 49%, respectively [26]. Patients from more deprived areas were significantly less likely to attend, and men less so than women.

The aim of this study was therefore to quantify the cost-effectiveness and equity impact of the NHS Health Check programme in Liverpool, comparing current implementation, targeted implementation, or their combination with structural interventions.

## Methods

The IMPACT_NCD_ model, a discrete-time stochastic microsimulation, was used to counterfactually simulate individual life courses in the scenarios. The model structure and validation is described in detail elsewhere [20,27,28]. We present, in detail, model inputs, model outputs, scenario specification, and additional results and validation in S1 Text. In brief, we set the simulation horizon to 2040 to enable this preventative intervention to have time to become effective, and we present the results for ages 30 to 84.

### Model inputs

We inputted data on demographics and projections (by age, sex, and English Index of Multiple Deprivation quintile group [QIMD]) for Liverpool into the IMPACT_NCD_ model to create a synthetic dynamic population of 200 million adults aged 30 to 84 at baseline. QIMD is a measure of relative area deprivation based on the Index of Multiple Deprivation [29]. According to this system, all Lower Super Output Areas in England (average population of 1,500) are ranked in order of increasing deprivation, based on 7 domains of deprivation: income; employment; health deprivation and disability; education, skills, and training; barriers to housing and services; crime and disorder; and living environment. Then, the QIMD is formed from the quintiles of the above index. Population exposure to 7 known CVD risks was extracted from the Health Survey for England (HSE) using a subsample of northwest England residents. The 7 risks were inadequate fruit and vegetable consumption, physical inactivity, smoking, high body mass index, hypertension, hypercholesterolaemia, and diabetes mellitus. Trends in these risks between 2002 and 2014 were projected to 2040, stratified by demographics, to estimate future exposure. We used the published relative risks from high-quality meta-analyses and cohort studies to link risk factor exposure with disease outcomes. Table 1 summarises the input sources for IMPACT_NCD_, and Table 2 presents the key modelling assumptions.

**Table 1:** IMPACT_NCD_ data sources.

Parameter	Outcome	Details	Comments	Source
Mortality rates	Deaths from nonmodelled causes	Mortality and midyear population estimates for England	Stratified by age, sex, QIMD, and cause of death. Years 2002–2013.	Data requested and obtained by the Office for National Statistics [30]
Population projections for Liverpool	Population size	Midyear population figures for Liverpool	Stratified by age and sex. Years 2014–2039. QIMD distribution was assumed to remain stable as in 2011. Population size for year 2040 was assumed the same as 2039.	Subnational population projections [31]
Exposure to risk factors	Exposure of individuals	HSE (northwest subsample)	Anonymised, individual-level datasets. Years 2001–2012.	HSE 2002–2014 [32–44]
RR for active smoking	CHD and stroke (ICD10: I20–I25 and I60–I69)	Re-analysis of American Cancer Society’s Cancer Prevention Study II. Prospective cohort study, 6 years of follow-up	Stratified by age and sex. Adjusted for age, race, education, marital status, ‘blue collar’ employment in most recent or current job, weekly consumption of vegetables and citrus fruit, vitamin (A, C, and E) use, alcohol use, aspirin use, body mass index, exercise, dietary fat consumption, hypertension, and diabetes at baseline.	Table 1 (Model B) in Ezzati and colleagues [45]
	Other mortality (except CHD and stroke)	Male British doctors prospective cohort study	Age-standardised	Table 1 in Doll and colleagues [46]
RR for ex-smoking	CHD (ICD10: I20–I25)	Meta-analysis. Multiple-adjusted pooled estimates from 19 prospective studies	Multiply-adjusted	Web Figure 8 in Huxley RR and colleagues [47]
	Stroke (ICD10 I60–I69)	The Framingham study. Prospective cohort study	Stroke risk decreased significantly by 2 years and was at the level of nonsmokers by 5 years after cessation of cigarette smoking. Therefore, we considered no risk for ex-smokers.	Wolf and colleagues [48]
RR for environmental tobacco smoking	CHD (ICD10: I20–I25)	Meta-analysis of 10 cohort and case-control studies	Adjusted for important CHD risk factors.	Table 3 (adjusted RR) in He and colleagues [49]
	Stroke (ICD10 I60–I69)	Meta-analysis of 20 prospective, case-control, and cross-sectional studies	Thirteen studies adjusted for important CHD risk factors. The overall effect of all 20 studies was used.	Figure 1 in Oono and colleagues [50]
RR for systolic blood pressure	CHD and stroke (ICD10: I20–I25 and I60–I69)	Meta-analysis of individual data from 61 prospective studies	Stratified by age and sex. Adjusted for regression dilution and total blood cholesterol and, where available, lipid fractions (HDL and non-HDL cholesterol), diabetes, weight, alcohol consumption, and smoking at baseline.	Figures 3 and 5 in Prospective Studies Collaboration [51]
RR for total cholesterol	CHD and stroke (ICD10: I20–I25 and I60–I69)	Meta-analysis of individual data from 61 prospective studies	Stratified by age and sex. Adjusted for regression dilution and age, sex, study, systolic blood pressure, and smoking.	Web Table 6 (fully adjusted) and Figure 3 in Prospective Studies Collaboration [52]
RR for body mass index	CHD and stroke (ICD10: I20–I25 and I60–I69)	Meta-analysis of 58 prospective studies	Stratified by age. Adjusted for age, sex, smoking status, systolic blood pressure, history of diabetes, and total and HDL cholesterol. We used the age gradient from the adjusted only for age, sex, and smoking status reported estimates.	Table 1 and Figure 2 in The Emerging Risk Factors Collaboration [53]
RR for diabetes mellitus	CHD and stroke (ICD10: I20–I25 and I60–I69)	Meta-analysis of 102 prospective studies	Stratified by age. Adjusted for age, smoking status, body mass index, and systolic blood pressure.	Figure 2 in The Emerging Risk Factors Collaboration [54]
	Other mortality (except CHD and stroke)	DECODE. A collaborative prospective study of 22 cohorts in Europe	Adjusted for body mass index, blood pressure, smoking, and serum cholesterol.	The DECODE Study Group [55]
RR for physical activity	CHD and stroke (ICD10: I20–I25 and I60–I69)	Meta-analysis of 18 cohort studies for CHD and 8 cohort studies for ischaemic stroke	Stratified by age and sex. Adjusted for measurement error, age, sex, smoking, blood pressure, and cholesterol.	Tables 10.19 and 10.20 in Bull and colleagues [56]
RR for fruit and vegetable consumption	CHD (ICD10: I20–I25)	Meta-analysis of 9 cohort studies	RR per portion of F&V. Multiply-adjusted.	Dauchet and colleagues [57]
	Stroke (ICD10: I60–I69)	Meta-analysis of 7 cohort studies	RR per portion of F&V. Multiply-adjusted.	Dauchet and colleagues [58]
Persistence with medication	Persistence to statins for primary prevention	Danish cohort study	No clear socioeconomic gradient was observed.	Wallach-Kildemoes and colleagues [59]
Adherence to medication	Persistence to statins for primary prevention	Danish cohort study	No clear socioeconomic gradient was observed.	Wallach-Kildemoes and colleagues [59]

**Abbreviations:** CHD, coronary heart disease; F&V, fruit and vegetable; HDL, high-density lipoprotein; HSE, Health Survey for England; RR, relative risk; QIMD, Index of Multiple Deprivation quintile group.

**Table 2:** IMPACT_NCD_ key assumptions and limitations.

Assumptions and limitations
Migration flows and social mobility were not considered in our estimates.
We assumed that the data sources that we used are genuinely representative of the Liverpool population.
We did not explicitly model alcohol consumption.
We assumed multiplicative risk effects for all risk factors and log-linear exposure–response relationship for the continuous ones.
We explicitly modelled hypertension, diabetes, CHD, and stroke. We defined CVD as the sum of CHD and stroke cases (deaths). We did not model other noncommunicable diseases that could potentially be affected by the modelled interventions.
We assumed that the observed trends in exposures and CVD mortality will continue in the future.
We assumed that trends in CHD and stroke incidence are attributable only to the modelled risk factor exposure trends.

**Abbreviations:** CHD, coronary heart disease; CVD, cardiovascular disease.

### Model outputs

The following list summarises model outputs:

1. CVD cases and deaths prevented or postponed by a modelled intervention, cumulatively over the simulated years.
2. Non-CVD deaths prevented or postponed by a modelled intervention, cumulatively over the simulated years. The model only considers smoking- and diabetes-related prevented or postponed non-CVD deaths.
3. QALYs gained because of a modelled intervention, cumulatively over the simulated years.
4. The net cost of a modelled intervention, cumulatively over the simulated years.
5. The incremental cost-effectiveness ratio (ICER) of a modelled intervention, as the ratio of cumulative net cost by cumulative QALYs gained (cost–utility analysis).
6. The net monetary benefit (NMB) assuming £20,000 willingness to pay.
7. The impact of an intervention on absolute and relative socioeconomic inequalities in health. We used the ‘absolute equity slope index’ and ‘relative equity slope index’—2 regression-based metrics—to measure the impact of the modelled interventions on absolute and relative socioeconomic health inequalities [27].

### Uncertainty and sensitivity analysis

Second-order Monte Carlo simulation was used to estimate 95% uncertainty intervals (UIs), propagating estimated uncertainty of inputs to the outputs. Many sources of uncertainty are shared between scenarios; therefore, between-scenario results are not statistically independent and covary to an extent. Therefore, a crossover between UIs for scenarios should not be seen as evidence against statistical significance (please refer to S1 Text for between-scenario comparisons). We present the probability estimates of each scenario being cost-effective (net cost of less than £20,000 per QALY gained), cost-saving (negative net cost), and equitable (reduces both absolute and relative socioeconomic inequalities in health). We defined a probability threshold of 80% to determine when—or whether—a scenario becomes cost-effective (or cost-saving, or equitable).

### Costs

We used local audit data to determine costs [25], while anonymised aggregated data from the local Clinical Commissioning Group were used to estimate prescription rates. The modelled costs were based on the payment that Liverpool City Council makes to GPs to provide the NHS Health Check programme [25]. This is £5.11 for an invitation and £13 to £19 per participant who undergoes a Health Check. The range of participation cost reflects that Liverpool City Council incentivises GP practices that achieve high uptake. Disease costs were drawn from NICE economic modelling and were separated by the first year of diagnosis, subsequent years, and fatal CVD events [60–63]. Deprivation weighting was used to match the deprivation profile of Liverpool, as there is evidence that there is a social gradient of costs [64]. Estimates assume that costs are equal for all ages and sexes, while myocardial infarction is used as a proxy for coronary heart disease (CHD). Non-CVD complications of diabetes are not included in cost estimates. Costs are inflated using the UK Treasury GDP deflator, November 2016 [65].

### Health-related quality of life

We searched for disease utility index score multipliers that used the Euroqol 5-dimension scale (EQ-5D), which is seen as the ‘gold standard’ for health technology assessment in the UK and is recommended by NICE [66]. The evidence base was used to determine baseline utility scores by age [67]. A multiplier of 0.778 was used for CHD [68], 0.629 for stroke [69], and 0.901 for diabetes [70]. Hypertension was not given a multiplier because there was no consistent evidence, especially considering the link between hypertension and other morbidities.

### Productivity losses

Productivity losses were estimated separately for CHD and stroke, accounting for both working years lost due to early mortality and sickness absence [71,72]. Prices were inflated using ONS data, then weighted based on Liverpool median earnings (95% of the median earnings for the UK). Indirect costs (such as informal care) were not included, and productivity losses were not included in the main cost estimations, which were from a health and social care perspective.

### Discounting

An annual discount rate of 3.5% was applied from 2016 to net costs and QALYs. Results from prior to 2016 were inversely discounted. This rate was selected based on guidance from the UK Treasury [73].

### Scenarios

In total, 20 scenarios were progressively developed through an iterative process with public health practitioners in Liverpool. Those scenarios included isolated improvements in coverage, uptake, prescription, referrals to lifestyle services, and some of their combinations. None were shown to be substantially better than the current implementation except the 5 scenarios we present in this study (for a detailed description and justification, please refer to S1 Text).

**(A) Current implementation.** In this scenario, we modelled the impact and costs assuming that the NHS Health Check programme continued unchanged in Liverpool throughout the modelled period to 2040. This assumed that annual coverage would remain at 13.8%, annual uptake would be 32.3%, and prescription rates would be 9.1% for low-risk, 25.8% for medium-risk, and 41.7% for high-risk participants. The costs were estimated at £5.11 per invitation and £13.28 per successful participation in NHS Health Check.

**(B) Current plus targeted.** In this scenario depicting a proportionate universalism approach, we modelled the impact and costs if the NHS Health Check programme was targeted toward individuals in the most deprived quintile, with increased coverage and uptake in this population while coverage and uptake in all other groups remain as the current implementation scenario. The coverage in the most deprived quintile is assumed to be 20%, with uptake at 66%. Crucially, in this scenario, we assume that the hypothetical recruitment strategy in the most deprived areas manages to attract participants with a higher cardiovascular risk profile than in scenario A. Prescription rates are unchanged from the current implementation scenario. The costs were estimated at £5.11 per invitation and £15.00 per successful participation in NHS Health Check. The increase in participation cost is in line with current Liverpool City Council practice to monetarily incentivise GP practices that achieve high uptake.

**(C) Optimal uptake and prescription rates.** In this scenario, we modelled the impact and costs if the NHS Health Check programme met the PHE requirements to invite 20% of the eligible population, and uptake was 66% [74]. Prescription rates in this scenario were set to 9.1% for low-risk, 80% for medium-risk, and 80% for high-risk participants to better reflect current prescription guidelines. In addition, we assumed highly effective lifestyle services. The costs were estimated at £5.11 per invitation and £15.00 per participant.

**(D) Current plus structural interventions.** This scenario modelled the impact and costs of the current implementation of NHS Health Check (scenario A) with the addition of structural interventions. The structural interventions were stricter tobacco control, an increase of fruit and vegetable consumption by a portion per day in 50% of individuals in the population, mandatory salt reformulation of processed foods, and an SSB tax of 20%. Their effectiveness was informed by published, mostly modelling studies [20,21,75–77].

**(E) Current plus targeted plus structural interventions.** This scenario modelled the impact and costs of the targeted implementation of NHS Health Check (scenario B) with the addition of structural interventions. The structural interventions are as in the previous scenario.

All results were reported following CHEERS guidelines (S1 CHEERS Checklist).

## Results

The model estimated that about 94% (95% UI: 93% to 96%) of CHD incidence can be attributed to the modelled risk factors in the least-deprived quintile group. This gradually increased with deprivation to reach 96% (95% UI: 94% to 97%) for the most-deprived quintile group. Similarly, for stroke, the proportions were 86% (95% UI: 81% to 90%) and 92% (95% UI: 89% to 94%) for the least- and most-deprived quintile groups, respectively.

### Effectiveness

Table 3 compares scenarios considering the number of CVD cases prevented and number of QALYs gained. The ‘current implementation’ was the worst-performing scenario for both outcomes and by both years (2030 and 2040). The ‘optimal implementation’ of Health Check performed better (estimated to prevent or postpone approximately 750 and 2,000 CVD cases by 2030 and 2040, respectively). However, that performance was dwarfed when structural interventions were added. The ‘targeted Health Check plus structural interventions’ was the best-performing scenario, estimated to prevent or postpone approximately 1,800 and 3,800 CVD cases by 2030 and 2040, respectively. Notably, even ‘current implementation plus structural interventions’ outperforms all programme-only scenarios.

**Table 3:** Comparison table of the effectiveness of the modelled scenarios. Ages 30 to 84. Parentheses contain 95% UIs. Results are rounded to the first 2 significant digits.

Model output	Scenario	By the year 2030	By the year 2040
Cumulative CVD cases prevented or postponed	Current (A)	290 (150 to 500)	570 (320 to 890)
	Current plus targeted (B)	530 (270 to 930)	1,200 (730 to 1,900)
	Optimal (C)	750 (400 to 1,300)	2,000 (1,400 to 2,900)
	Current plus structural (D)	1,600 (1,000 to 2,300)	3,300 (2,400 to 4,200)
	Current plus targeted plus structural (E)	1,800 (1,100 to 2,700)	3,800 (2,900 to 5,000)
Cumulative net QALYs gained (discounted)	Current (A)	57 (−130 to 310)	220 (−110 to 660)
	Current plus targeted (B)	85 (−200 to 490)	500 (−82 to 1,300)
	Optimal (C)	310 (−110 to 960)	1,700 (700 to 3,100)
	Current plus structural (D)	2,400 (1,100 to 4,300)	7,000 (4,600 to 10,000)
	Current plus targeted plus structural (E)	2,400 (1,000 to 4,500)	7,200 (4,700 to 10,000)

**Abbreviations:** CVD, cardiovascular disease; QALY, quality-adjusted life year; UI, uncertainty interval.

### Cost-effectiveness

**Fig 1:**
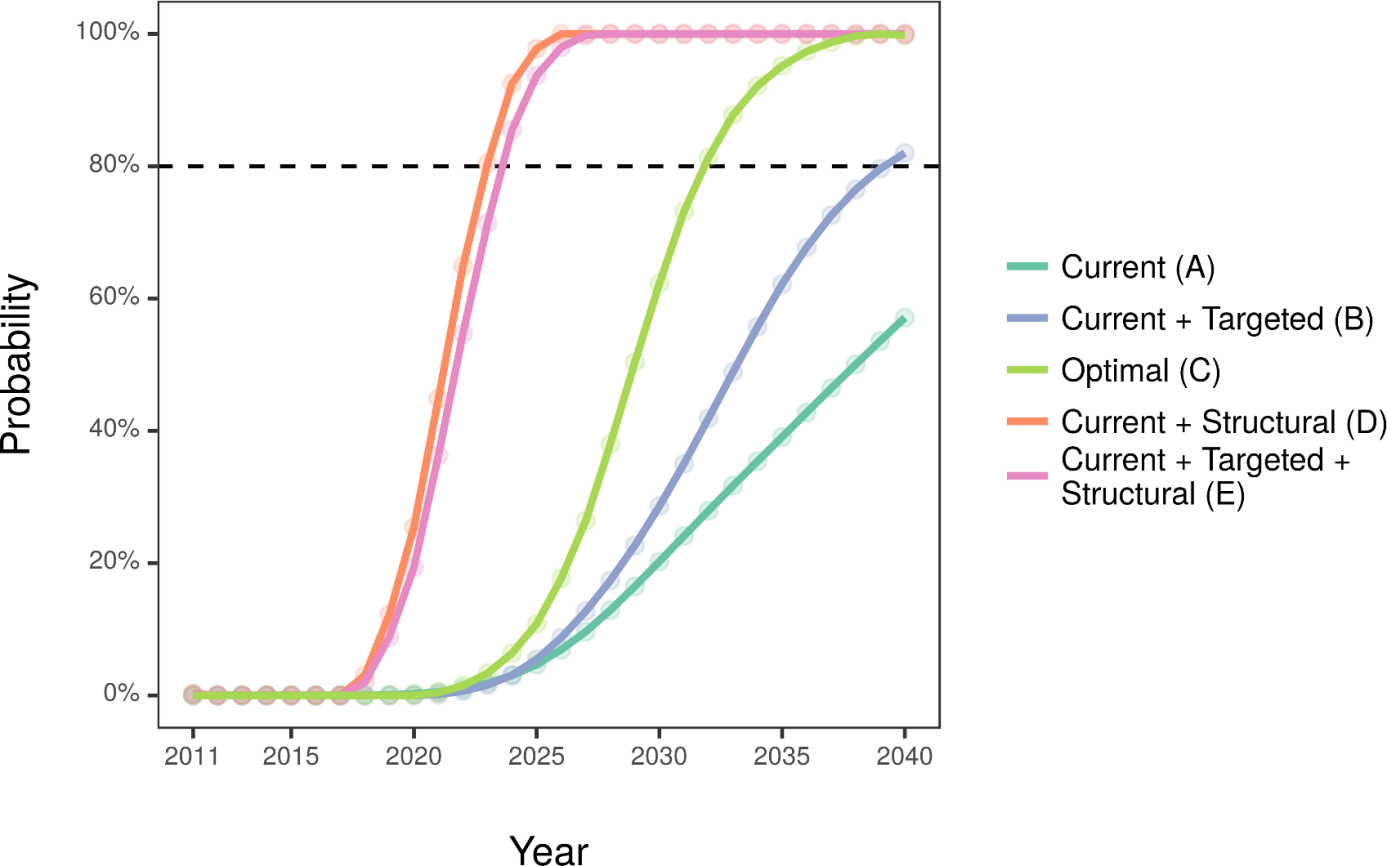
Annual probability of the modelled scenarios to be cost-effective. Willingness to pay £20,000 per QALY. QALY, quality-adjusted life year.

**Fig 2:**
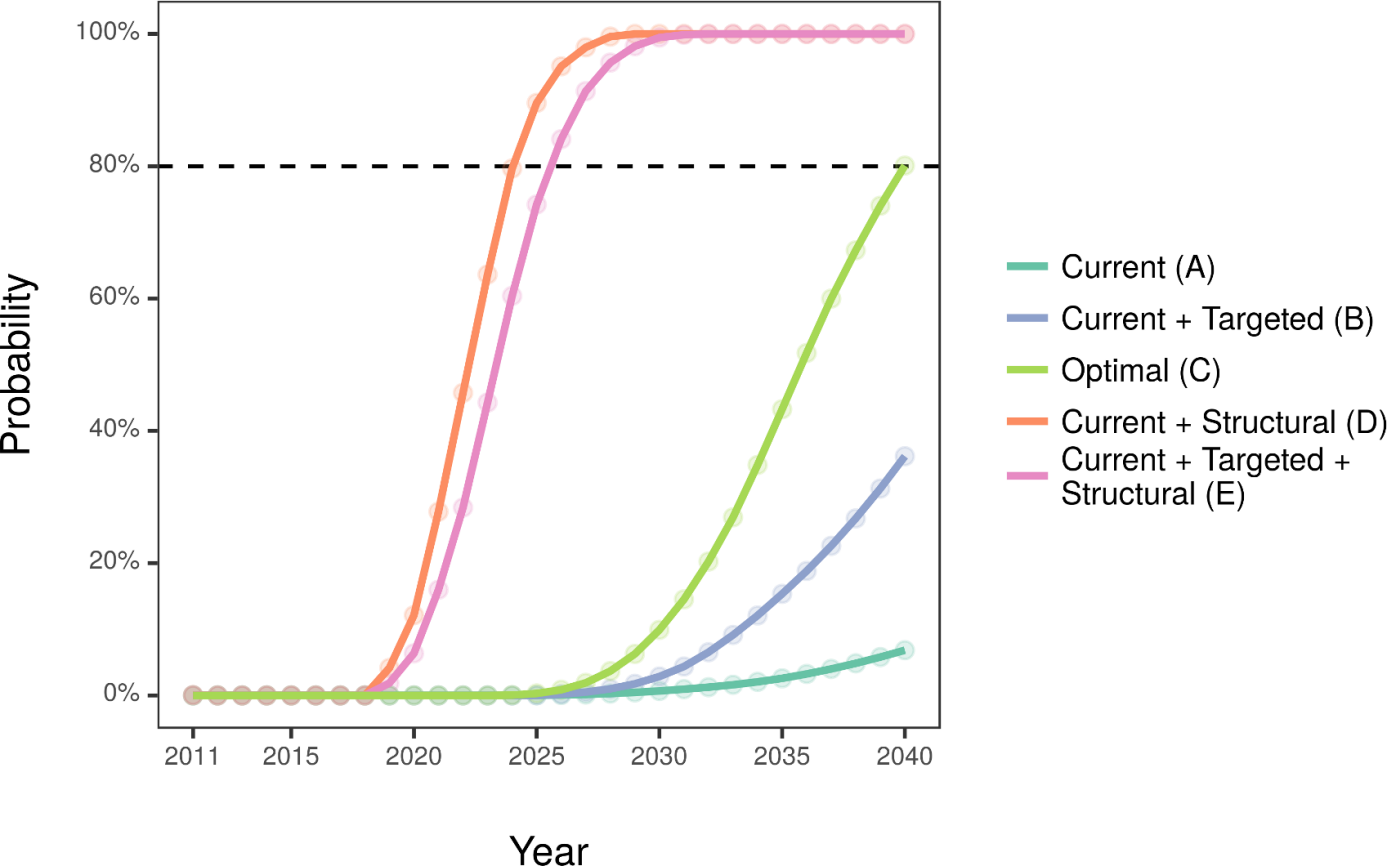
Annual probability of the modelled scenarios to be cost-saving.

Table 4 and Fig 1 show cost-effectiveness analysis. The current implementation was unlikely to become cost-effective before 2040, while the targeted implementation would only pass the 80% threshold by 2040. The optimal implementation would pass the threshold by 2032. Comparatively, the addition of structural interventions means that scenarios D and E might become cost-effective by 2023, almost a decade earlier. Fig 2 shows that current and targeted implementations are unlikely to be cost-saving by 2040, while optimal implementation might reach the threshold by 2040. The current implementation with structural interventions could reach cost-saving fastest—by 2024—while targeted and structural approaches are likely to achieve cost-saving by 2026.

**Table 4:** Comparison table of the cost-effectiveness of the modelled scenarios. Negative net costs are essentially savings. Ages 30 to 84. Parentheses contain 95% UIs. Results are rounded to the first 2 significant digits.

Model output	Scenario	By the year 2030	By the year 2040
Cumulative net cost (discounted £million)	Current (A)	4.0 (1.1 to 6.2)	3.4 (−1.5 to 6.9)
	Current plus targeted (B)	4.7 (−0.1 to 7.9)	1.3 (−8.6 to 7.5)
	Optimal (C)	3.9 (−2.8 to 8.2)	−4.2 (−18.0 to 4.3)
	Current plus structural (D)	−13.0 (−28.0 to −3.7)	−35.0 (−60.0 to −19.0)
	Current plus targeted plus structural (E)	−11.0 (−27.0 to −1.7)	−35.0 (−63.0 to −18.0)
Cumulative ICER (discounted £/QALY)	Current (A)	21,000 (−650,000 to 730,000)	11,000 (−270,000 to 320,000)
	Current plus targeted (B)	14,000 (−450,000 to 540,000)	1,500 (−91,000 to 100,000)
	Optimal (C)	9,700 (−170,000 to 190,000)	−2,400 (−6,500 to 5,700)
	Current plus structural (D)	−5,200 (−8,400 to −2,600)	−5,100 (−7,400 to −3,200)
	Current plus targeted plus structural (E)	−4,600 (−7,700 to −1,400)	−5,000 (−7,400 to −3,100)
Cumulative NMB (discounted £million)	Current (A)	−3.0 (−8.3 to 5.0)	0.9 (−8.6 to 14.0)
	Current plus targeted (B)	−3.0 (−11.0 to 10.0)	8.8 (−8.6 to 34.0)
	Optimal (C)	2.5 (−10.0 to 22.0)	38.0 (10.0 to 79.0)
	Current plus structural (D)	62.0 (27.0 to 110.0)	180.0 (120.0 to 250.0)
	Current plus targeted plus structural (E)	60.0 (23.0 to 110.0)	180.0 (120.0 to 270.0)

**Abbreviation:** ICER, incremental cost-effectiveness ratio; NMB, net monetary benefit; QALY, quality-adjusted life year; UI, uncertainty interval.

### Equity

**Fig 3:**
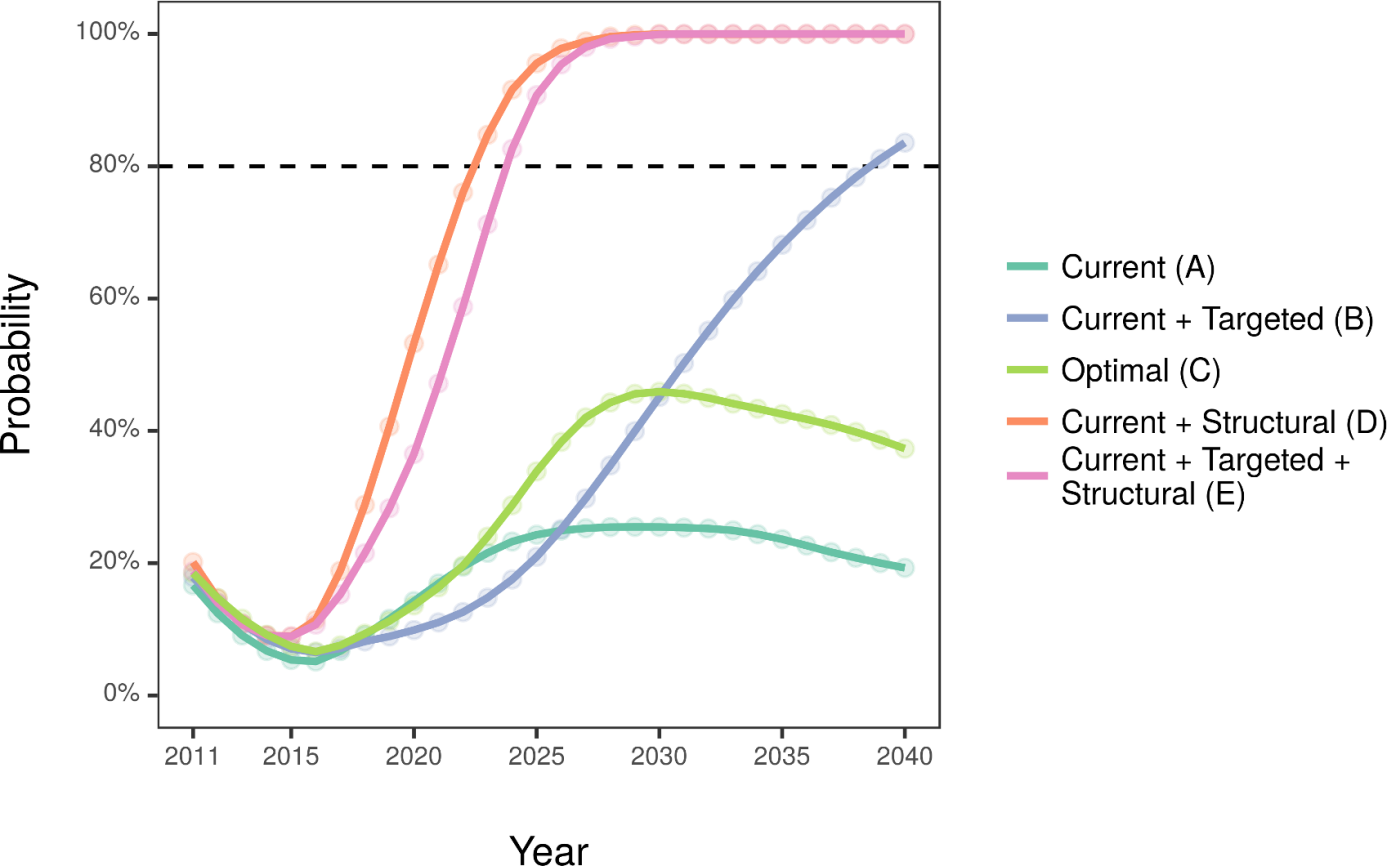
Annual probability of the modelled scenarios to be equitable. We defined equitable as reducing both absolute and relative socioeconomic inequalities in health.

Table 5 and Fig 3 display findings concerning health inequalities. All 3 programme-only scenarios showed a decrease in absolute inequality—modest for the current implementation and substantial for the optimal implementation. When considering relative inequalities, only the targeted implementation was associated with an improvement in equity, crossing the 80% threshold by 2039. By contrast, the structural intervention scenarios both resulted in much larger improvements in both absolute and relative inequalities. Both would cross the 80% threshold before 2025.

**Table 5:** Comparison table of the equity of the modelled scenarios. Positive values represent a reduction in inequalities and vice versa. Ages 30 to 84. Parentheses contain 95% UIs. Results are rounded to the first 2 significant digits.

Model output	Scenario	By the year 2030	By the year 2040
Reduction in absolute socioeconomic health inequalities	Current (A)	150 (−570 to 1,100)	600 (−660 to 2,300)
	Current plus targeted (B)	410 (−1,000 to 2,600)	2,900 (−360 to 7,700)
	Optimal (C)	1,300 (−340 to 3,900)	7,200 (3,100 to 13,000)
	Current plus structural (D)	13,000 (5,800 to 22,000)	37,000 (24,000 to 52,000)
	Current plus targeted plus structural (E)	13,000 (5,300 to 23,000)	38,000 (25,000 to 55,000)
Reduction in relative socioeconomic health inequalities	Current (A)	−24 (−230 to 130)	−76 (−330 to 140)
	Current plus targeted (B)	11 (−150 to 200)	120 (−110 to 400)
	Optimal (C)	−2.1 (−270 to 210)	−50 (−440 to 270)
	Current plus structural (D)	550 (160 to 1,100)	1,200 (630 to 1,900)
	Current plus targeted plus structural (E)	550 (130 to 1,200)	1,300 (670 to 2,000)

**Abbreviation:** UI, uncertainty interval.

### Productivity

All 5 scenarios would produce productivity savings by 2040 (Table 6). These savings would be largest for the targeted Health Check plus structural interventions (scenario E), with savings of approximately £23 million above the current implementation. This was over 5 times greater savings than for targeted implementation (scenario B) alone and 4 times greater than the savings increase for optimal implementation.

**Table 6:** CHD, stroke, and CVD net productivity discounted cost by the year 2040 (30 years). Ages 30 to 64. Parentheses contain 95% UIs. Negative values represent savings. Results are rounded to the first 2 significant digits.

Scenario	CHD net productivity cost (£million)	Stroke net productivity cost (£million)	Total CVD net productivity cost (£million)
Current (A)	−2.0 (−4.1 to −0.84)	−0.74 (−1.7 to −0.18)	−2.7 (−5.5 to −1.2)
Current plus targeted (B)	−5.2 (−10.0 to 2.6)	−1.7 (−3.7 to −0.69)	−6.8 (−14.0 to −3.6)
Optimal (C)	−5.5 (−11.0 to −2.9)	−2.6 (−4.9 to −1.2)	−8.1 (−15.0 to −4.5)
Current plus structural (D)	−13.0 (−20.0 to −8.0)	−9.8 (−15.0 to −6.1)	−22.0 (−34.0 to −15.0)
Current plus targeted plus structural (E)	−15.0 (−25.0 to −9.7)	−10.0 (−17.0 to −6.7)	−26.0 (−41.0 to −17.0)

**Abbreviations:** CHD, coronary heart disease; CVD, cardiovascular disease; UI, uncertainty interval.

## Discussion

Our study suggests that simply continuing the current implementation of the Health Check programme in Liverpool is unlikely to be cost-effective or equitable, even when modelled 2 decades into the future. If implementation of the programme met optimal PHE recommendations for uptake and prescribing [74], then cost-effectiveness might be achieved by 2030 and cost-savings achieved by 2040 for the Liverpool population. However, it would not improve relative health inequalities. Conversely, a targeted approach towards the most deprived (those at the highest risk) would likely improve equity by 2039 but only reach cost-effectiveness by 2040.

Moreover, our findings may have wider implications nationally and beyond. NHS Health Check is an intervention that targets high-risk individuals. Theoretically, these interventions are more effective when the risk is concentrated in some subgroups of the population [78]. Historically, Liverpool is a city with concentrated deprivation and concentrated CVD risk that translates consistently to worse CVD burden and outcomes compared to the national average [79]. Therefore, we expect the effectiveness and cost-effectiveness of NHS Health Check—even considering optimal implementation—to be worse elsewhere than in Liverpool.

These findings add to the results of a systematic review by Lee and colleagues in 2017 [9]. Analysing the evidence for the cost-effectiveness of population-wide CVD screening, the review found that there was a lack of robust evidence to support the implementation of such screening. As a result, the authors recommended that further evidence is required to identify the cost-effectiveness of such screening, and different delivery models should be examined, such as targeted implementation or population-wide interventions. This modelling study examines these issues directly and provides the evidence recommended.

The addition of structural interventions addressing smoking, diet, and an SSB tax provides a stark contrast. These scenarios would almost certainly be cost-effective, cost-saving, and equitable and reach these 80% thresholds much more quickly. This is unsurprising when remembering Rose’s paradigm about sick individuals and sick populations [80].

The UK Department of Health estimated that the ICER for NHS Health Check would be under £3,000 per QALY gained [6]. However, our findings suggest the ICER would be substantially higher than this by 2030, regardless of implementation. Furthermore, substantial improvements to the delivery and implementation of NHS Health Check would be required to achieve an ICER under £3,000 per QALY gained by 2040. Our estimates are comparable to those from Crossan and colleagues suggesting that optimal implementation would reduce ICERs, perhaps to less than £10,000 (versus £23,000 or more for typical implementation) [10]. The different estimates of these studies are due to differences in the modelled NHS Health Check implementation—and thus effectiveness—and wider differences in the modelling assumptions overall (i.e., assumptions regarding the future burden of CVD or use of different simulation horizons).

### Public health implications

These results from our IMPACT_NCD_ model may be of particular interest to local commissioners. Public health interventions have generally been found to be cost-saving [81], thus NHS Health Check appears to represent a comparatively expensive approach to prevention [82]. However, the NHS Health Check programme is currently a statutory requirement for local governments in England. Given this and the major pressures on local-authority budgets [83], the need to improve the cost-effectiveness of prevention programmes with the addition of cost-saving interventions is becoming increasingly clear.

The UK government introduced a sweetened drinks industry levy (SSB tax) in April 2018 [84]. However, other population strategies to reduce CVD could be added. Rather than wait for further action at the national level, we would advocate that local governments take the initiative in implementing population health-improvement strategies. Liverpool did that in the past. In 2004, the local government took the initiative to make Liverpool a smoke-free city. This local initiative was instrumental in the passage of the relevant national law 2 years later [85].

A number of cities across the United States, such as Boston and Oklahoma [86,87], have engaged in collective approaches to encourage healthier diets and weight loss. New York City implemented a number of approaches to improve fruit and vegetable consumption, particularly among low-income individuals [88]. These approaches included financial support to reduce cost barriers, incentives to improve access, support to improve supply and demand for healthy food, improving food quality in different organisations (including schools and childcare centres), and individual support programmes. This combined approach was associated with small but significant improvements in fruit and vegetable consumption. The legal and political feasibility of similar local public health policies for English cities is largely unexplored. Moreover, devolution deals such as those in Liverpool and Manchester may give local governments additional power to implement more structural preventive policies.

### Strengths and limitations

To our knowledge, IMPACT_NCD_ is the first dynamic stochastic microsimulation model to estimate cost-effectiveness and equity of the NHS Health Check programme at a city level. Focusing on the city level allowed us to use real-world data on disease rates, costs, and programme success that are not easily available at the national level. In addition, the choice of Liverpool—a city in which the concentration of CVD risk theoretically would favour NHS Health Check—allows the derivation of useful analogies for other areas.

There are, however, a number of limitations. First, health-related quality of life decrements were assumed equal across all socioeconomic groups. However, when considering CVD, people from lower socioeconomic backgrounds have significantly reduced quality of life compared to higher socioeconomic backgrounds. This is likely to underestimate our cost-effectiveness and equity estimates for scenarios B and E [89]. Second, the costs for NHS Health Check only consider the prices paid by the Liverpool City Council. We have assumed that these costs are consistent and have not considered the potential effects of saturation or diminishing returns. The costs of medications for CHD, stroke, diabetes, and hypertension are included in the broad healthcare unit cost estimates. Only the costs of statins for people with no other diseases are not explicitly included, but these costs are small, at around £20 to £40 per patient per annum. Furthermore, we did not include opportunity costs, resulting in the potential for the Health Check to displace more effective health interventions [90]. Third, while most of the structural policies we modelled are incremental improvements on existing policies, their political feasibility is unclear in the current environment. Fourth, our model only considers CVD and diabetes mellitus. Both NHS Health Check and the structural interventions we modelled can potentially reduce the burden of other noncommunicable diseases, too.

## Conclusions

Our results suggest that current NHS Health Check implementation appears neither equitable nor cost-effective for local authorities. Optimal administration and implementation might result in better value for money in the next few decades. However, the addition of structural interventions could substantially reduce CVD risks, improve equity, and generate cost savings within short time-scales.

## Supporting information

S1 CHEERS Checklist CHEERS reporting checklist. (DOCX)

S1 Text Supplementary technical appendix. (PDF)

## Abbreviations

CHD: coronary heart disease
CVD: cardiovascular disease
EQ-5D: Euroqol 5-dimension scale
GP: General Practice
HDL: high-density lipoprotein
HSE: Health Survey for England
ICER: incremental cost-effectiveness ratio
NHS: National Health Service
NMB: net monetary benefit
PHE: Public Health England
QALY: quality-adjusted life year
QIMD: Index of Multiple Deprivation quintile group
SSB: sugar-sweetened beverage
UI: uncertainty interval
