# Supplementary material for: Future cost-effectiveness and equity of the NHS Health Check cardiovascular disease prevention programme: Microsimulation modelling using data from Liverpool, UK
Source: PLoS Med. 2018 May 29;15(5):e1002573. doi: 10.1371/journal.pmed.1002573 (PMC5973555; doi:10.1371/journal.pmed.1002573)
Supplement: S1 Text — (PDF) [file pmed.1002573.s002.pdf]

Supplementary Technical Appendix

This appendix has been provided by the authors to give readers additional information about their work.

Supplement to: **Future cost-effectiveness and equity of the NHS Health Checks cardiovascular disease prevention programme: microsimulation modelling using data from Liverpool, UK**

Table of Contents

Supplementary Technical Appendix 1

Table of Contents 2

SUMMARY OF METHODS 4

- The model 4
  - Model inputs 4
  - Model outputs 5
  - Model calibration and validation 6
  - Key modelling assumptions and limitations 8
- Uncertainty 9
- Costs 24
- Health-related quality of life 26
  - Uncertainty around health-related quality of life 26
- Productivity losses 27
- Discounting 27
- The scenarios 27

SCENARIO RESULTS 30

- Baseline 30
- Scenario A: Current implementation 31
- Scenario B: Increased annual coverage to 20% and uptake to 66% in the most deprived fifth of national IMD from 2017 37
- Scenario C: Optimal implementation from 2017 39
- Scenario D: Current implementation + structural interventions 41
- Scenario E: Targeted implementation + all structural interventions 44
- Between scenario comparisons (probability of superiority) 45

CVD BURDEN PROJECTIONS FOR LIVERPOOL 52

References 58

SUMMARY OF METHODS

The model

$IMPACT_{NCD}$ simulates the life course of individuals under counterfactual scenarios based on widely accepted epidemiological principles. It is a discrete-time stochastic dynamic microsimulation. The technical specifications of the model have been published elsewhere.[1–3] For this study, we calibrated $IMPACT_{NCD}$ to simulate the population of the Liverpool City Council area. In the following paragraphs, we provide a high-level description of the model inputs and outputs.

Model inputs

We populated the $IMPACT_{NCD}$ model with data detailing the Liverpool city demographics and demographic projections (by age, sex, and national Index of Multiple Deprivation quintile groups, QIMD). We used the subsample of Health Survey for England (HSE) participants living in Northwest England to extract current and past population exposures to seven known cardiovascular disease (CVD) risk factors; inadequate fruit & vegetable consumption, physical inactivity, smoking, excess body mass index (BMI), hypertension, hypercholesterolaemia, and diabetes mellitus, for years 2002 to 2014. Then, we projected past risk factor exposures trends to the year 2040 stratified by age, sex, and QIMD to estimate future population exposures. Subsequently, the different scenarios were modelled through their effect on these seven risk factors for selected individuals or the whole population. Every simulated year a new cohort of synthetic individuals enter the simulation at the age of 30. The size of the cohort is informed by the official population projection for Liverpool.[4] The QIMD distribution in the cohort is assumed to be that of 2011.[*]

Furthermore, $IMPACT_{NCD}$ requires coronary heart disease (CHD) and stroke incidence, prevalence, and case-fatality rates for the initial simulated year, stratified by age, sex, and QIMD. There are no reliable sources for these inputs that could directly inform the model, locally or nationally. To overcome this, we used DisMod II, a multistate life table model that can estimate the incidence, prevalence, mortality, case-fatality, and remission rates of a disease, when information about at least three of these indicators is available.[5] We informed DisMod II with national disease-specific mortality rates by the Office for National Statistics (ONS), and self-reported prevalence of CHD and stroke from HSE for the

[*] The relative QIMD distribution in the population of Liverpool remained remarkably stable between 2004 and 2014. Hence, we assumed that this will continue in the future. Migration flows are not considered in the simulation.

year 2011. We used DisMod II incidence, prevalence, and case-fatality rate estimates for CHD and stroke to inform $IMPACT_{NCD}$. $IMPACT_{NCD}$ then estimate disease incidence and prevalence based on the projections of risk factor exposures and disease-specific mortality based on projections of CVD case fatality rates and non-CVD* mortality rates.

Model outputs

$IMPACT_{NCD}$ outputs that have been used for this report are:

1. CVD cases and deaths prevented or postponed by a modelled intervention, cumulatively over the simulated period.
2. Non-CVD deaths prevented or postponed by a modelled intervention, cumulatively over the simulated period. The model only considers smoking and diabetes-related non-CVD prevented or postponed deaths.
3. Quality-adjusted life years (QALYs) gained because of a modelled intervention, cumulatively over the simulated period.
4. The net cost of a modelled intervention, cumulatively over the simulated period.
5. ICER of a modelled intervention, as the ratio of cumulative net cost by cumulative QALYs, gained (cost-utility analysis or CUA).
6. The net monetary benefit (NMB) assuming £20,000 willingness to pay.
7. The impact of an intervention on absolute and relative socioeconomic inequalities in health. We used the 'absolute equity slope index' and 'relative equity slope index'; two regression-based metrics, to measure the impact of the modelled interventions on absolute and relative socioeconomic health inequalities. They are inspired by the slope index of inequality (SII) and the relative index of inequality (RII);[6] however, instead of directly measuring inequalities in a population, as SII and RII do, they measure the impact of an intervention on existing inequalities.

For all these outputs, $IMPACT_{NCD}$ estimates 95% uncertainty intervals (UI) using a second-order Monte Carlo simulation. This probabilistic sensitivity analysis of the model, propagates the estimated uncertainty of the model inputs, to the model outputs.

To better express and communicate the uncertainty of the model we also plot the probability of a scenario to be cost-effective cost saving and equitable for every simulated year (2011 to 2040). We defined 'cost-effective' as 'having net cost less than £20,000 per QALY gained'; 'cost saving' as 'having negative net cost'; and 'equitable' as 'reducing both absolute and relative socioeconomic inequalities in CVD'. When a scenario has more than 80% probability to be cost-effective, cost saving, or equitable

* We only model CHD (ICD10 I20-I25) and stroke (ICD10 I60-I69) and no other diseases of the CVD spectrum. For simplicity when we report model outputs, we use the term CVD for the sum of CHD and stroke cases (representing ICD10 I20-I25 plus I60-I69).

after a certain year 20XX we report that ‘the scenario is likely to become cost-effective (or cost saving or equitable) from year 20XX.’

Model calibration and validation

We used ONS reported CHD, stroke, and all-cause mortality in Liverpool for years 2011 to 2015 and ages 30 to 84 to calibrate our model. The pre-calibration mortality estimates were lower than the observed ones, especially for the most deprived quintile. This underestimation can be explained mainly by two reasons:

1) The case fatality rates we used in the model were representative of the mean case fatality rate within each deprivation quintile. Liverpool most deprived areas (QIMD 4 and 5) are more deprived than the mean QIMD 4 and 5 areas nationally. Therefore, it is safe to assume that the true case fatality rates in Liverpool are higher than the estimates we used. To account for this, we inflated CHD and stroke case fatality rate by 5% and 12%, respectively, for the most deprived fifth of national IMD. Moreover, we inflated the non-CVD mortality rate by 17% for the most deprived fifth of national IMD and by 10% for the second most deprived fifth.
2) CVD mortality rates in England are declining because of declining incidence rates and improvements in survival of patients with CVD.[7] $IMPACT_{NCD}$ simulates declines in CVD incidence by modelling trends in CVD risk factor exposures, but improvements in patient survival were not modelled pre-calibration. To model future improvements in patient survival, we assumed 5% (relative) annual decline of CHD case fatality rate and 2% (relative) for stroke.

The following graphs compare the CHD, stroke, and all-cause $IMPACT_{NCD}$ estimated post-calibration mortality, with the observed ones by fifth of deprivation for ages 30 to 84 and years 2011 to 2015, in Liverpool (Figures A-C). In general, the graphs support that the post-calibration model replicates CVD and all-cause mortality, adequately for the aims of this study.

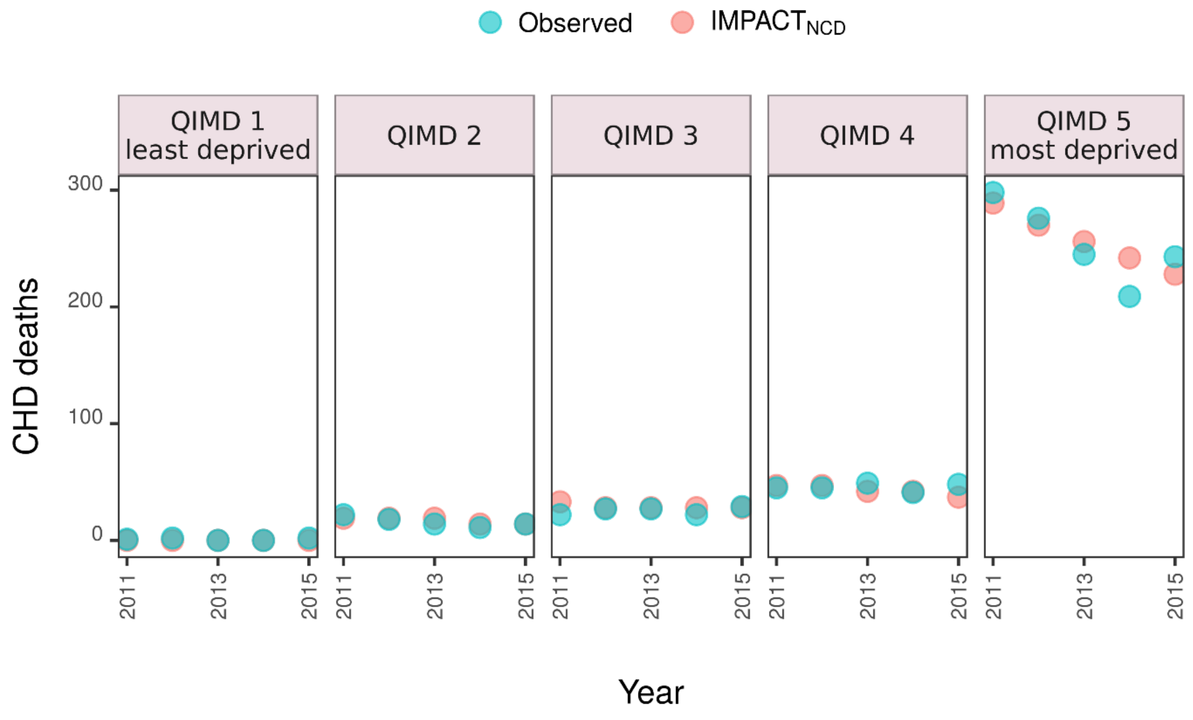

Figure A Comparison of post-calibration $IMPACT_{NCD}$ estimated CHD mortality with the observed one by fifth of deprivation for ages 30 to 84 and years 2011 to 2015, in Liverpool.

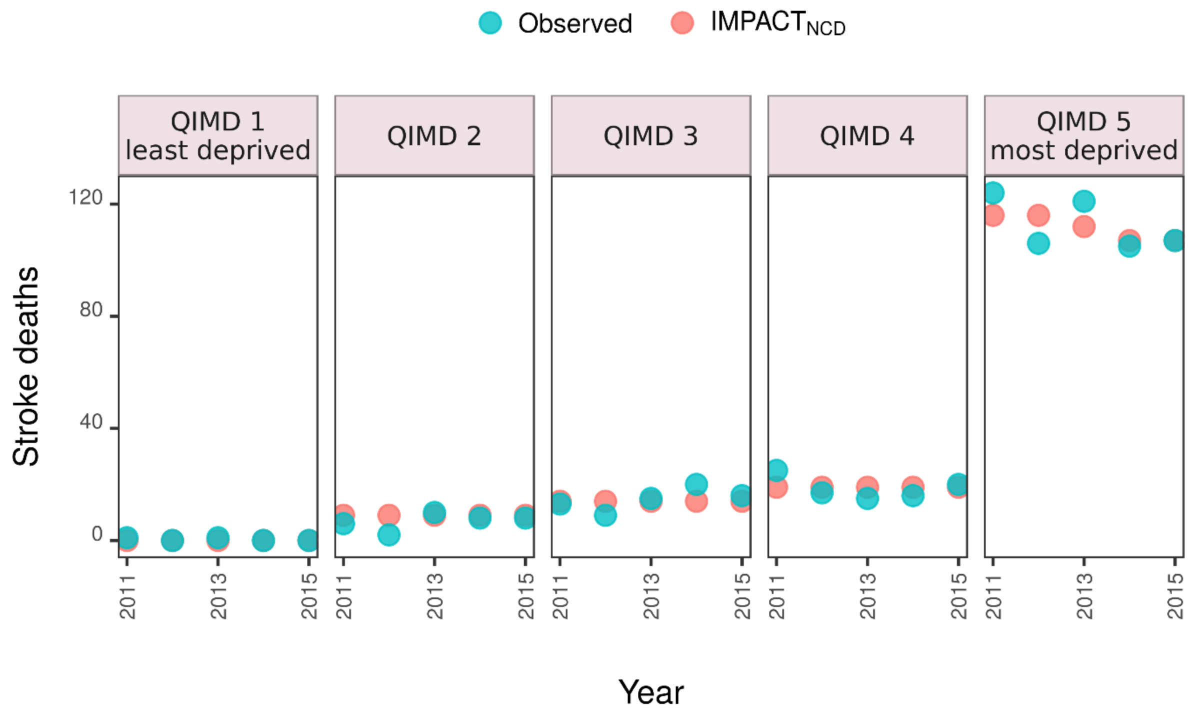

Figure B Comparison of post-calibration $IMPACT_{NCD}$ estimated stroke mortality with the observed one by fifth of deprivation for ages 30 to 84 and years 2011 to 2015, in Liverpool.

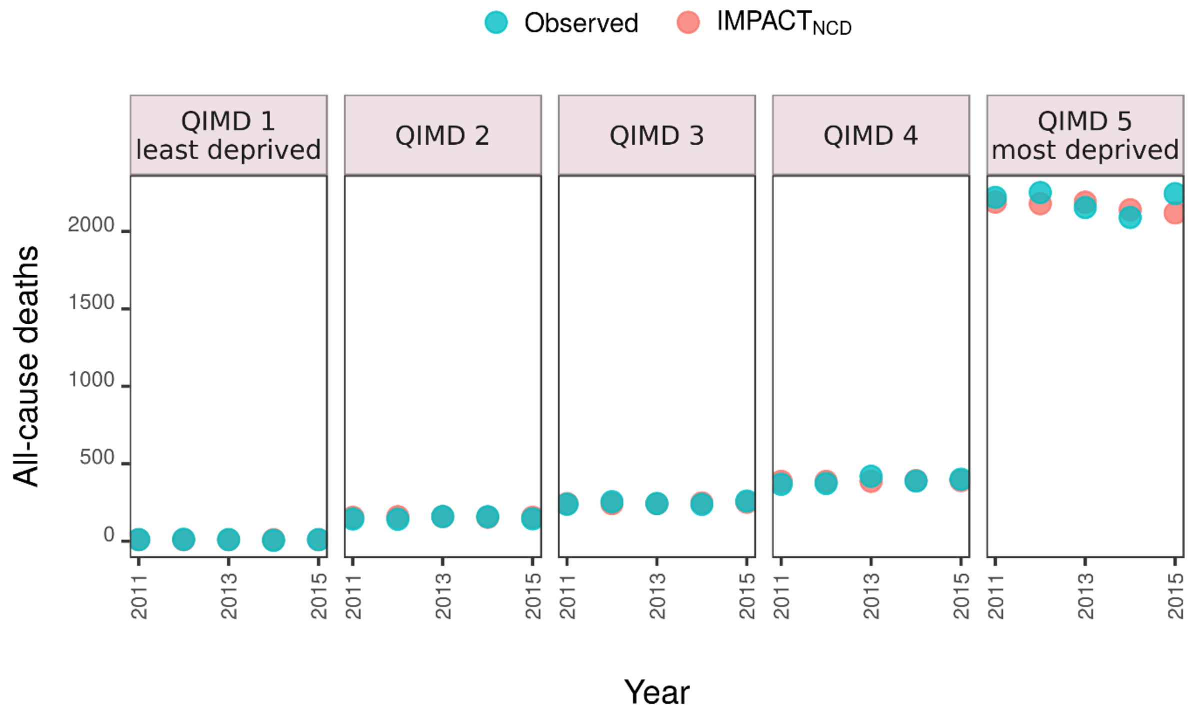

Figure C Comparison of post-calibration IMPACT$_{NCD}$ estimated all-cause mortality with the observed one by fifth of deprivation for ages 30 to 84 and years 2011 to 2015, in Liverpool.

Key modelling assumptions and limitations

In our modelling approach, we were obliged to make a number of assumptions and simplifications. We present the most important ones below:

1. Migration flows and social mobility were not considered in our estimates.
2. We assumed that the data sources that we have used are genuinely representative of Liverpool population.
3. We did not explicitly model alcohol consumption.
4. We assumed multiplicative risk effects for all risk factors and log-linear exposure-response relationship for the continuous ones.
5. We explicitly modelled hypertension, diabetes, CHD and stroke. We defined CVD as the sum of CHD and stroke cases (deaths). We did not model other non-communicable diseases that could potentially be affected by the modelled interventions.
6. We assumed that the observed trends in exposures and CVD mortality will continue in the future.
7. We assumed that trends in CHD and stroke incidence are attributable only to the modelled risk factor exposure trends.

These assumptions were common for all the scenarios we modelled. We report the key scenario-specific assumptions with each scenario in the next chapter.

Uncertainty

IMPACT$_{\mathbf{NCD}}$ implements a 2nd order Monte Carlo approach to estimate uncertainty intervals (UI) for each scenario.[8,9] Each simulation runs 2000 times. For each iteration, a different set of input parameters is used, by sampling from the respective distributions (Table A)* of input parameters, and a different sample of the synthetic population is drawn. Therefore, the scenarios are 'paired'. For instance, the *n*th iteration of all scenarios runs with the same set of input parameters and on the same synthetic population sample for all of them. This explains why overlapping UIs are not evidence against statistical significance. Tables I – N summarise between scenarios comparisons

The framework allows stochastic uncertainty, parameter uncertainty, and individual heterogeneity to be reflected in the reported UI. The following example illustrates the different types of uncertainty that were considered in IMPACT$_{NCD}$. Let us assume that the annual risk for CHD is 5%. If we apply this risk to all individuals and randomly draw from a Bernoulli distribution with p = 5% to select those who will manifest CHD, we only consider stochastic uncertainty. If we allow the annual risk for CHD to be conditional on individual characteristics (i.e. age, sex, exposure to risk factors), then individual heterogeneity is considered. Finally, when the uncertainty of the relative risks due to sampling errors is considered in the estimation of the annual risk for CHD, the parameter uncertainty is considered. From these three types of uncertainty, only the parameter uncertainty can be reduced from better studies in the future.

Due to lack of information and for computational efficiency, different types of uncertainty are considered in different IMPACT$_{NCD}$ processes. Specifically, stochastic uncertainty is included in all processes, although the algorithm cancels out stochastic uncertainty that is shared among all scenarios. Individual heterogeneity is modelled for disease incidence and mortality calculations, as well as invitation and participation to an NHS Health Check, and potential treatment after the participation. Similarly, it is considered for the modelled structural policies. Heterogeneity was also considered for disease costs, as we considered different costs by QIMD and different costs for the first episode of CHD and stroke.

* We assumed log-normal distributions for relative risks and hazard ratios, normal distributions for coefficients of linear regression equations, generalized beta of the second kind for costs and PERT distributions for other parameters. Specifically for relative risks and hazard ratios, the distributions were bounded above 1 when the mean was above 1 and vice versa. This was to avoid run Monte Carlo iterations in which well-accepted risk factors were treated as protective factors.

Finally, parameter uncertainty we considered the sampling error of the relative risks for CHD, stroke, and any-other-cause mortality risk factors. We used the reported relative risks and their confidence intervals to inform log-normal distributions. Similarly, we considered the sampling error of the quality of life decrements used to calculate QALY for which a normal distribution was assumed. Finally, we considered the uncertainty around disease costs. We fitted generalized beta of the second kind distributions assuming the 0.2 percentile to be 80% of the central estimate, the median the central estimate, and the 0.8 percentile 120% of the central estimate. A study by Jones et al. found that this distribution was the most accurate for health care cost data.[10] Health care costs per person typically follow a skewed distribution with a small number of people having high costs.[11] All the distributions that have been used as inputs are summarised in Table A.

The structure of the model is grounded on fundamental epidemiological ideas and well-established causal pathways; therefore, we considered this type of uncertainty relatively small and did not study it. However, mortality from each of the modelled diseases and the any-other cause is calculated serially, one modelled disease at a time. To avoid bias that this approach might introduce, the order of the modelled diseases in each mortality estimation is randomized.

Table A Distributions that were used as inputs for the simulations. Numbers are rounded.

Variable	Sex	Ages	Distribution
**Relative risks for CHD**			
Active smoking[12 table 1 model B]	Men	30 - 44	Log-Normal (mean = ln(5.51), sd = ln(12.3 / 5.51) / 1.96)
		45 - 59	Log-Normal (mean = ln(3.04), sd = ln(3.48 / 3.04) / 1.96)
		60 - 69	Log-Normal (mean = ln(1.88), sd = ln(2.08 / 1.88) / 1.96)
		70 - 79	Log-Normal (mean = ln(1.44), sd = ln(1.63 / 1.44) / 1.96)

Variable	Sex	Ages	Distribution
	Women	30 - 44	Log-Normal (mean = ln(2.26), sd = ln(6.14 / 2.26) / 1.96)
		45 - 59	Log-Normal (mean = ln(3.78), sd = ln(4.62 / 3.78) / 1.96)
		60 - 69	Log-Normal (mean = ln(2.53), sd = ln(2.87 / 2.53) / 1.96)
		70 - 79	Log-Normal (mean = ln(1.68), sd = ln(1.93 / 1.68) / 1.96)
		80 - 84	Log-Normal (mean = ln(1.38), sd = ln(1.77 / 1.38) / 1.96)
Ex-Smoking[13 web-figure 8]	Men	30 - 84	Log-Normal (mean = ln(1.25), sd = ln(1.32 / 1.25) / 1.96)
	Women	30 - 84	Log-Normal (mean = ln(1.2), sd = ln(1.34 / 1.2) / 1.96)
Environmental tobacco smoking[14 table 3, adjusted RR]	Both	30 - 84	Log-Normal (mean = ln(1.26), sd = ln(1.38 / 1.26) / 1.96)

Variable	Sex	Ages	Distribution
Systolic blood pressure[15 figure 5]	Men	30 - 49	Log-Normal (mean = ln(0.5), sd = ln(0.54 / 0.5) / 1.96)
		50 - 59	Log-Normal (mean = ln(0.5), sd = ln(0.52 / 0.5) / 1.96)
		60 - 69	Log-Normal (mean = ln(0.55), sd = ln(0.57 / 0.55) / 1.96)
		70 - 74	Log-Normal (mean = ln(0.62), sd = ln(0.64 / 0.62) / 1.96)
		80 - 84	Log-Normal (mean = ln(0.69), sd = ln(0.73 / 0.69) / 1.96)
	Women	30 - 49	Log-Normal (mean = ln(0.4), sd = ln(0.49 / 0.4) / 1.96)
		50 - 59	Log-Normal (mean = ln(0.49), sd = ln(0.54 / 0.49) / 1.96)
		60 - 69	Log-Normal (mean = ln(0.5), sd = ln(0.61 / 0.5) / 1.96)
		70 - 74	Log-Normal (mean = ln(0.55), sd = ln(0.58 / 0.55) / 1.96)

Variable	Sex	Ages	Distribution
		80 - 84	Log-Normal (mean = ln(0.64), sd = ln(0.68 / 0.64) / 1.96)
Total cholesterol[16 web-table 6]	Both	30 - 49	Log-Normal (mean = ln(0.49), sd = ln(0.52 / 0.49) / 1.96)
		50 - 59	Log-Normal (mean = ln(0.62), sd = ln(0.65 / 0.62) / 1.96)
		60 - 69	Log-Normal (mean = ln(0.74), sd = ln(0.76 / 0.74) / 1.96)
		70 - 74	Log-Normal (mean = ln(0.84), sd = ln(0.86 / 0.84) / 1.96)
		80 - 84	Log-Normal (mean = ln(0.87), sd = ln(0.9 / 0.87) / 1.96)
BMI[17 table 1 and figure 2]	Both	30 - 59	Log-Normal (mean = ln(1.21), sd = ln(1.28 / 1.21) / 1.96)
		60 - 69	Log-Normal (mean = ln(1.06), sd = ln(1.12 / 1.06) / 1.96)

Variable	Sex	Ages	Distribution
Diabetes mellitus[18 figure 2]	Both	40 - 59	Log-Normal (mean = ln(2.51), sd = ln(2.8/ 2.51) / 1.96)
		60 - 69	Log-Normal (mean = ln(2.01), sd = ln(2.26/ 2.01) / 1.96)
		70 - 84	Log-Normal (mean = ln(1.78), sd = ln(2.05/ 1.78) / 1.96)
Physical activity[19 table 10.19]	Both	30 - 69	No active days: Log-Normal (mean = ln(1.71), sd = ln(1.85/ 1.71) / 1.96)
			1 – 4 active days: Log-Normal (mean = ln(1.44), sd = ln(1.62/ 1.44) / 1.96)
		70 - 79	No active days: Log-Normal (mean = ln(1.5), sd = ln(1.61/ 1.5) / 1.96)
			1 – 4 active days: Log-Normal (mean = ln(1.31), sd = ln(1.48/ 1.31) / 1.96)
		80 - 84	No active days: Log-Normal (mean = ln(1.4), sd = ln(1.41/ 1.4) / 1.96)
			1 – 4 active days: Log-Normal (mean = ln(1.2), sd = ln(1.35/ 1.2) / 1.96)

Variable	Sex	Ages	Distribution
Fruit & Vegetable intake[20]			Log-Normal (mean = ln(0.96), sd = ln(1.0.99/ 0.96) / 1.96)
**Relative risks for stroke**			
Active smoking[12 table 1 model B]	Men	30 - 59	Log-Normal (mean = ln(3.12), sd = ln(4.64 / 3.12) / 1.96)
		60 - 69	Log-Normal (mean = ln(1.87), sd = ln(2.44 / 1.87) / 1.96)
		70 - 79	Log-Normal (mean = ln(1.39), sd = ln(1.77 / 1.39) / 1.96)
	Women	30 - 59	Log-Normal (mean = ln(4.61), sd = ln(6.37 / 4.61) / 1.96)
		60 - 69	Log-Normal (mean = ln(2.81), sd = ln(3.58 / 2.81) / 1.96)
		70 - 79	Log-Normal (mean = ln(1.95), sd = ln(2.45 / 1.95) / 1.96)
Environmental tobacco smoking [21 figure 1]	Both	30 - 84	Log-Normal (mean = ln(1.25), sd = ln(1.38 / 1.25) / 1.96)

Variable	Sex	Ages	Distribution
Systolic blood pressure[15 figure 3]	Men	30 - 49	Log-Normal (mean = ln(0.33), sd = ln(0.38 / 0.33) / 1.96)
		50 - 59	Log-Normal (mean = ln(0.34), sd = ln(0.37 / 0.34) / 1.96)
		60 - 69	Log-Normal (mean = ln(0.41), sd = ln(0.44 / 0.41) / 1.96)
		70 - 74	Log-Normal (mean = ln(0.48), sd = ln(0.51 / 0.48) / 1.96)
		80 - 84	Log-Normal (mean = ln(0.68), sd = ln(0.75 / 0.68) / 1.96)
	Women	30 - 49	Log-Normal (mean = ln(0.41), sd = ln(0.49 / 0.41) / 1.96)
		50 - 59	Log-Normal (mean = ln(0.45), sd = ln(0.5 / 0.45) / 1.96)
		60 - 69	Log-Normal (mean = ln(0.47), sd = ln(0.51 / 0.47) / 1.96)
		70 - 74	Log-Normal (mean = ln(0.53), sd = ln(0.56 / 0.53) / 1.96)

Variable	Sex	Ages	Distribution
		80 - 84	Log-Normal (mean = ln(0.65), sd = ln(0.71 / 0.65) / 1.96)
Total cholesterol[16 figure 3]	Both	40 - 49	Log-Normal (mean = ln(0.87), sd = ln(1 / 0.87) / 1.96)
		50 - 59	Log-Normal (mean = ln(0.91), sd = ln(0.97 / 0.91) / 1.96)
		60 - 69	Log-Normal (mean = ln(0.93), sd = ln(0.97 / 0.93) / 1.96)
BMI[17 table 1 and figure 2]	Both	30 - 59	Log-Normal (mean = ln(1.18), sd = ln(1.26 / 1.18) / 1.96)
		60 - 69	Log-Normal (mean = ln(1.08), sd = ln(1.15 / 1.08) / 1.96)
Diabetes mellitus[18 figure 2]	Both	40 - 59	Log-Normal (mean = ln(3.74), sd = ln(4.58/ 3.74) / 1.96)
		60 - 69	Log-Normal (mean = ln(2.06), sd = ln(2.58/ 2.06) / 1.96)

Variable	Sex	Ages	Distribution
		70 - 84	Log-Normal (mean = ln(1.8), sd = ln(2.27/ 1.8) / 1.96)
Physical activity[19 table 10.20]	Both	30 - 69	No active days: Log-Normal (mean = ln(1.53), sd = ln(1.79/ 1.53 / 1.96)
		70 - 79	No active days: Log-Normal (mean = ln(1.38), sd = ln(1.6/ 1.38) / 1.96)
		80 - 84	No active days: Log-Normal (mean = ln(1.24), sd = ln(1.45/ 1.24) / 1.96)
Fruit & Vegetable[22]			Log-Normal (mean = ln(0.95), sd = ln(0.97/ 0.95) / 1.96)
**Health-related quality of life scores**			
Event free[23]	Both	30 - 34	Normal (mean = 0.93, sd = 0.005)
		35 - 44	Normal (mean = 0.91, sd = 0.007)
		45 - 49	Normal (mean = 0.869, sd = 0.011)
		50 - 54	Normal (mean = 0.848, sd = 0.011)
		55 - 59	Normal (mean = 0.826, sd = 0.012)

Variable	Sex	Ages	Distribution
		60 - 64	Normal (mean = 0.805, sd = 0.012)
		65 - 69	Normal (mean = 0.784, sd = 0.012)
		70 - 74	Normal (mean = 0.763, sd = 0.012)
		75 - 79	Normal (mean = 0.741, sd = 0.015)
		80 - 84	Normal (mean = 0.72, sd = 0.015)
CHD (exclusive)[24]	Both	30 - 84	Normal (mean = 0.778, sd = 0.038)
Stroke (exclusive)[25]	Both	30 - 84	Normal (mean = 0.629, sd = 0.04)
Diabetes mellitus (exclusive)[26]	Both	30 - 84	Normal (mean = 0.901, sd = 0.035)
**Mortality costs**			
CHD (exclusive, year of the first event)[27 Table 61]	Both	30 - 84	Betaprime (shape 1 = 41.0, shape 2 = 19101.6, scale = 760483.2)
Stroke (exclusive, consequent years)[27 Table 61]	Both	30 - 84	Betaprime (shape 1 = 41.1, shape 2 = 9406.1, scale = 1097352)
**Disease costs (QIMD 1, least deprived)**[28]			
CHD (exclusive, year of the first event)[27 Table 61]	Both	30 - 84	Betaprime (shape 1 = 41.1, shape 2 = 9569.7, scale = 670329.2)
CHD (exclusive, consequent years)[27 Table 61]	Both	30 - 84	Betaprime (shape 1 = 41.2, shape 2 = 7775.9, scale = 179675.4)
Stroke (exclusive, year of the first event)[29 Table 4]	Both	30 - 84	Betaprime (shape 1 = 646.5, shape 2 = 44.8, scale = 579.1)

Variable	Sex	Ages	Distribution
Stroke (exclusive, consequent years)[29 Table 4]	Both	30 - 84	Betaprime (shape 1 = 41.3, shape 2 = 4974.7, scale = 454849.0)
Hypertension (exclusive, year of the first event)[30 Table 113]	Both	30 - 84	Betaprime (shape 1 = 41.0, shape 2 = 26766.3, scale = 76304.6)
Hypertension (exclusive, consequent years)[30 Table 113]	Both	30 - 84	Betaprime (shape 1 = 41.0, shape 2 = 71193.6, scale = 109392.0)
Diabetes mellitus (exclusive)	Both	30 - 84	Betaprime (shape 1 = 41.0, shape 2 = 38041.9, scale = 476655.8)
**Disease costs (QIMD 2)**[28]			
CHD (exclusive, year of the first event)[27 Table 61]	Both	30 - 84	Betaprime (shape1 = 41.4, shape 2 = 4421.6, scale = 324669.7)
CHD (exclusive, consequent years)[27 Table 61]	Both	30 - 84	Betaprime (shape1 = 41.1, shape 2 = 15732.4, scale = 384819)
Stroke (exclusive, year of the first event)[29 Table 4]	Both	30 - 84	Betaprime (shape1 = 41.3, shape 2 = 5229.2, scale = 1142317.3)
Stroke (exclusive, consequent years)[29 Table 4]	Both	30 - 84	Betaprime (shape1 = 41.3, shape 2 = 5402.9, scale = 522248.3)
Hypertension (exclusive, year of the first event)[30 Table 113]	Both	30 - 84	Betaprime (shape1 = 41, shape 2 = 77253.2, scale = 231764)
Hypertension (exclusive, consequent years)[30 Table 113]	Both	30 - 84	Betaprime (shape1 = 41, shape 2 = 145292.9, scale = 233950.3)
Diabetes mellitus (exclusive)	Both	30 - 84	Betaprime (shape1 = 41.1, shape 2 = 23767.5, scale = 313555.7)
**Disease costs (QIMD 3)**[28]			
CHD (exclusive, year of the first event)[27 Table 61]	Both	30 - 84	Betaprime (shape1 = 41.1, shape 2 = 10615.1, scale = 796773.3)
CHD (exclusive, consequent years)[27 Table 61]	Both	30 - 84	Betaprime (shape1 = 41.1, shape 2 = 14108.6, scale = 350143.4)

Variable	Sex	Ages	Distribution
Stroke (exclusive, year of the first event)[29 Table 4]	Both	30 - 84	Betaprime (shape1 = 41.4, shape 2 = 4779.3, scale = 1056488.3)
Stroke (exclusive, consequent years)[29 Table 4]	Both	30 - 84	Betaprime (shape1 = 41.4, shape 2 = 6211, scale = 607867.3)
Hypertension (exclusive, year of the first event)[30 Table 113]	Both	30 - 84	Betaprime (shape1 = 41, shape 2 = 111406.1, scale = 339716)
Hypertension (exclusive, consequent years)[30 Table 113]	Both	30 - 84	Betaprime (shape1 = 41, shape 2 = 126111.9, scale = 206132.7)
Diabetes mellitus (exclusive)	Both	30 - 84	Betaprime (shape1 = 41, shape 2 = 16922.6, scale = 226984.9)
**Disease costs (QIMD 4)**[28]			
CHD (exclusive, year of the first event)[27 Table 61]	Both	30 - 84	Betaprime (shape1 = 41.1, shape 2 = 9763, scale = 762923.9)
CHD (exclusive, consequent years)[27 Table 61]	Both	30 - 84	Betaprime (shape1 = 41, shape 2 = 17674.9, scale = 456988.7)
Stroke (exclusive, year of the first event)[29 Table 4]	Both	30 - 84	Betaprime (shape1 = 41.3, shape 2 = 4270.7, scale = 984688.9)
Stroke (exclusive, consequent years)[29 Table 4]	Both	30 - 84	Betaprime (shape1 = 41.4, shape 2 = 7184.1, scale = 732236.7)
Hypertension (exclusive, year of the first event)[30 Table 113]	Both	30 - 84	Betaprime (shape1 = 41, shape 2 = 104838.7, scale = 332469.2)
Hypertension (exclusive, consequent years)[30 Table 113]	Both	30 - 84	Betaprime (shape1 = 41, shape 2 = 41471.3, scale = 70772.7)
Diabetes mellitus (exclusive)	Both	30 - 84	Betaprime (shape1 = 41, shape 2 = 31892.3, scale = 446154.5)
**Disease costs (QIMD 5, most deprived)**[28]			

Variable	Sex	Ages	Distribution
CHD (exclusive, year of the first event)[27 Table 61]	Both	30 - 84	Betaprime (shape1 = 41.1, shape 2 = 9763, scale = 762923.9)
CHD (exclusive, consequent years)[27 Table 61]	Both	30 - 84	Betaprime (shape1 = 41, shape 2 = 17674.9, scale = 456988.7)
Stroke (exclusive, year of the first event)[29 Table 4]	Both	30 - 84	Betaprime (shape1 = 41.3, shape 2 = 4270.7, scale = 984688.9)
Stroke (exclusive, consequent years)[29 Table 4]	Both	30 - 84	Betaprime (shape1 = 41.4, shape 2 = 7184.1, scale = 732236.7)
Hypertension (exclusive, year of the first event)[30 Table 113]	Both	30 - 84	Betaprime (shape1 = 41, shape 2 = 104838.7, scale = 332469.2)
Hypertension (exclusive, consequent years)[30 Table 113]	Both	30 - 84	Betaprime (shape1 = 41, shape 2 = 41471.3, scale = 70772.7)
Diabetes mellitus (exclusive)	Both	30 - 84	Betaprime (shape1 = 41, shape 2 = 31892.3, scale = 446154.5)
**NHS Health Checks and variations**			
Atorvastatin effect (proportional reduction in total cholesterol)[31,32]	Both	40 - 84	Normal (mean = 0.32, sd = 0.014)
Persistence with medication[33]	Both	40 - 84	PERT (min = 0.5, mode = 0.8, max = 1, shape = 4)
Adherence to medication[33]	Both	40 - 84	PERT (min = 0.3, mode = 0.7, max = 1, shape = 4)
**Structural policies**			
SSB tax effect (QIMD 1, least deprived)[34 Table 8]	Both	30 - 49	Normal (mean = -0.08, sd = -0.05)

Variable	Sex	Ages	Distribution
		50 - 84	Normal (mean = -0.02, sd = -0.05)
SSB tax effect (QIMD 2 - 4)[34 Table 8]	Both	30 - 49	Normal (mean = -0.05, sd = -0.02)
		50 - 84	Normal (mean = 0.03, sd = 0.07)
SSB tax effect (QIMD 5, most deprived)[34 Table 8]	Both	30 - 49	Normal (mean = -0.06, sd = -0.01)
		50 - 84	Normal (mean = 0, sd = 0.04)
Stricter tobacco control (QIMD 1, least deprived)[35]	Men	30 - 84	PERT (min = 0.05, mode = 0.1, max = 0.21, scale = 4)
	Women	30 - 84	PERT (min = 0.04, mode = 0.1, max = 0.22, scale = 4)
Stricter tobacco control (QIMD 2)[35]	Men	30 - 84	PERT (min = 0.07, mode = 0.12, max = 0.2, scale = 4)
	Women	30 - 84	PERT (min = 0.06, mode = 0.12, max = 0.2, scale = 4)
Stricter tobacco control (QIMD 3)[35]	Men	30 - 84	PERT (min = 0.08, mode = 0.14, max = 0.2, scale = 4)
	Women	30 - 84	PERT (min = 0.07, mode = 0.14, max = 0.2, scale = 4)
Stricter tobacco control (QIMD 4)[35]	Men	30 - 84	PERT (min = 0.08, mode = 0.15, max = 0.2, scale = 4)
	Women	30 - 84	PERT (min = 0.08, mode = 0.15, max = 0.2, scale = 4)
Stricter tobacco control (QIMD 5, most deprived)[35]	Men	30 - 84	PERT (min = 0.09, mode = 0.19, max = 0.2, scale = 4)

Variable	Sex	Ages	Distribution
	Women	30 - 84	PERT (min = 0.08, mode = 0.19, max = 0.21, scale = 4)
Mandatory salt reformulation[2]	Both	30 - 84	Table too long to be included here. Please download the file with the parameters for the PERT distributions that were used from https://github.com/ChristK/IMPACTncd_Liverpool/blob/master/Scenarios/salt_reform_effect.csv

Costs

The modelled costs for NHS Health Checks were based on Liverpool City Council's payments to GPs for NHS Health Checks. This was £5.11 (per invited individual) and £13.00 - £19.00 per participant, where a higher payment was paid to practices that achieved uptake above 60% or 80%.

The most recent target for local authorities set by Public Health England (PHE) was to invite 20% of their target population annually and achieve an uptake rate of at least 66% each year.[36] The original target was 80% of people per annum invited to complete a health check.

The costs of disease (CHD, stroke, hypertension and diabetes) were drawn from economic modelling carried out for NICE, which is generally based on the best economic evidence that is available at the time in England. We considered separate costs for year one (first year after being diagnosed), subsequent years, and fatal CVD events which reflected higher costs in the final year of life. We did not include costs for non-CVD deaths and disease. Stroke costs are from an NHS perspective and include rehabilitation but not ongoing social care costs. Costs were weighted for deprivation (Table B) as there is good evidence that costs for the same disease show a social gradient. The weighting for deprivation was based on data from Charlton et al. who found that average disease costs vary by QIMD.[28] Apart from the weighting for deprivation, disease costs were not otherwise specifically weighted for Liverpool.

Table B Long-term condition costs from Charlton et al.[28]

QIMD	Cost per year - one morbidity (£)	Cost ratio relative to least deprived
1 Least deprived	744	1
2	785	1.05
3	797	1.07

4	830	1.11
5 Most deprived	917	1.23

Table C Disease costs used in the model.

Disease	Event Type	Original costs	Cost Year	2016 costs	Source	Table in the report
Ischemic Heart Disease	Non-fatal event – year of event	£2,274	2000	£3,088	NICE (2015) NG28[27]	Table 61
Ischemic Heart Disease	Non-fatal event – subsequent years	£751	2000	£1,020	NICE (2015) NG28[27]	Table 61
Stroke	Non-fatal event – year of event	£8,274	2009	£9,162	NICE (2010a) PH25[29]	Table 4
Stroke	Non-fatal event – subsequent years	£3,660	2009	£4,053	NICE (2010a) PH25[29]	Table 4
Diabetes type 2 treatment and management without complications	All years	£514	2011	£551	Hex et al. (2012)[37]	Table 2
Hypertension	Non-fatal event – year of event	£115	2010	£125	NICE (2011) CG127[30]	Table 113
Hypertension	Non-fatal event – subsequent years	£62	2010	£67	NICE (2011) CG127[30]	Table 113
Myocardial Infarction	Fatal event – year of event	£1,152	2000	£1,565	NICE (2015) NG28[27]	Table 61
Stroke	Fatal event – year of event	£3,383	2000	£4,594	NICE (2015) NG28[27]	Table 61

The disease costs we have are averages, and it is assumed that disease costs are the same for all age and sex groups (Table C). The cost of CHD deaths is based on costs of myocardial infarction deaths. The cost of multimorbidity is assumed to be the sum of costs of individual diseases. We have not specifically included costs of diabetes complications, except for stroke and CHD which are included as separate diseases; other complications like amputations or kidney failure were not included. We inflated all costs to 2016 using UK Treasury GDP inflator tables from November 2016. We did not use the PSSRU hospital & community health services index because it only goes back to 2004 and some of the costs predate this.

Health-related quality of life

Health-related quality of life (HRQOL) scores are used to calculate QALYs which are calculated as HRQOL multiplied by time in years. In the UK, the gold standard recommended by NICE is to use EQ-5D (Euroqol five dimension) utility index scores that are based on population preferences. Baseline health state utility index scores by age were drawn from the study by Kind et al.[23] Utility was applied based on multipliers for disease states. For CHD, a multiplier of 0.778 (standard error 0.038) was used from a study by Mistry et al.[24] There is a lack of health state utility scores for an 'average' individual with CHD. The CHD multiplier was the average of the values for myocardial infarction, stable angina, and unstable angina. These multipliers were all quite similar to each other (0.760, 0.808, and 0.769 respectively). For stroke, a multiplier of 0.629 (standard error 0.040) was assumed from a meta-analysis of quality of life estimates for stroke by Tengs and Lin.[25] For diabetes, a multiplier of 0.901 (standard error 0.035) was assumed from Clarke et al.[26] In our model we assumed that there was no utility decrement for hypertension alone; the evidence is not consistent about this as the majority of people with hypertension also have other diseases. However, in the model, when individuals have hypertension it increases the risk of other diseases which would cause a decrease in utility.

We assumed that utility associated with diseases is the same across IMD quintile groups. It would still be the case that average utility is lower in people from more deprived groups as they have a higher risk of disease, but we are not assuming an additional utility decrement for the effect of deprivation.

Uncertainty around health-related quality of life

There are three main uncertainties around health state utility values. Firstly, around the model and the sample that was used to produce the indices. In the UK for the EQ-5D, the indices were based on a representative sample of the population using the time trade-off method. Citizens were given health scenario vignettes and asked how much time they would trade off to spend a shorter amount of time in a better health state instead of a longer time in a poorer health state. The second level of uncertainty is around the mean health state utility index value for a given condition (or age, gender, deprivation category) which would be driven by the sample size. So a small sample may pick up people whose CVD is more or less severe than the general population in that category, whereas as the sample gets bigger, the standard error gets smaller and tends towards zero, and the average should tend towards the population average. The standard errors in our sources are quite small which indicates a high degree of certainty around the mean utility decrements for the disease groups. The third level of uncertainty is individual-level variation as described by the standard deviation. As the sample size gets bigger, this may reduce, but with a large sample size, the standard deviation will tend towards the

true population standard deviation. In our model, we have included the uncertainty around the mean decrement as measured by the standard error only.

Productivity losses

Workplace productivity losses for CHD were estimated using data from Liu et al., which included estimates of friction-adjusted employment productivity losses based on working years lost through early mortality, and certified incapacity days.[38] Productivity losses for stroke were estimated using data from Saka et al., which included income lost due to mortality and morbidity which was combined with the prevalence of stroke in people aged under 65 to get a unit cost.[39] These estimates were inflated to 2016 prices using the ratio of average weekly earnings data from ONS and then weighted for Liverpool based on median weekly earnings for Liverpool which were 95% of the UK average. The costs were estimated for men and women combined.

Other indirect costs such as informal care or out of pocket expenses were not included. Productivity losses were not included in the main cost per QALY and net monetary benefit calculations as we took a healthcare perspective for the main results.

Discounting

The costing year used was 2016. Costs and benefits that occur after 2016 were discounted by 3.5% per annum in line with NICE and UK Treasury guidance. For models runs which produced results before 2016, benefits that had occurred before 2016 were inversely discounted to 2016.

All the scenarios were discussed and co-developed with the Liverpool City Council Public Health team. Most of the scenarios are focusing on identifying and mapping areas of improvement in the current implementation of NHS Health Checks (i.e. better coverage, uptake, prescription rate, referrals to lifestyle services) that could make NHS Health Checks more effective, cost-effective and equitable. Two scenarios model the combination NHS Health Checks with structural interventions targeting diet and smoking, feasible at the national level, but that could also be supported by local action and public health leadership.

The scenarios

In total, 20 scenarios were progressively developed through an iterative process with public health practitioners in Liverpool. Those scenarios included isolated improvements in coverage, uptake, prescription, and referrals to lifestyle services and some of their combinations. None showed to be substantially better than the current implementation, except the five scenarios we included in this study and we summarise in Table D. We used the recent audit by Jones et al. on the local

implementation of NHS Health Checks and costs.[40] We also used aggregated anonymised data from Liverpool CCG about the risk profile of NHS Health Checks participants, and prescription rates after a health check to inform relevant model parameters. The Liverpool City Council's payment scheme to GPs for NHS Health Checks attendances incentivises GPs that achieve higher uptake of NHS Health Checks. Therefore, in the modelled scenarios that we assumed an increase in NHS Health Check uptake we also assumed an increase in the cost per participant from £13.28 to £15.00. All the scenarios were compared with the 'baseline' scenario that assumes no implementation of NHS Health Checks. The current implementation scenario starts in 2011. For all other scenarios, we assumed that the modelled change will happen from 2017 onwards by modifying the current implementation scenario. We simulated up to the year 2040 (simulation horizon).

Table D Summary of the modelled scenarios.

	**Scenario**	**Main assumptions**	**Policy costs**
A	Current	Coverage: 13.8%	£5.11 per invitation
		Uptake: 32.3%	£13.28 per participant
		Prescription rate: 9.1% (low risk) 25.8% (middle risk) 41.7% (high risk)	
B	Targeted	Coverage: 20% for the most deprived national IMD fifth and 13.8% for all other fifths	£5.11 per invitation<br>£15.00 per participant
		Uptake: 66% for the most deprived national IMD fifth and 32.3% for all other fifths	
		Prescription: 9.1% (low risk) 25.8% (middle risk) 41.7% (high risk)	
		Assumes the risk profile of participants from the most deprived fifth is similar to the risk profile of the population in the most deprived fifth.	
C	Optimal	Coverage: 20%	£5.11 per invitation<br>£15.00 per participant
		Uptake: 66%	
		Prescription: 9.1% (low risk) 80% (middle risk) 80% (high risk)	
		Increased referrals to highly effective lifestyle services	

	Scenario	Main assumptions	Policy costs
D	Current implementation with the addition of structural interventions	Coverage: 13.8%<br>Uptake: 32.3%<br>Prescription rate: 9.1% (low risk) 25.8% (middle risk) 41.7% (high risk)<br>Salt mandatory reformulation<br>SSB tax rate: 20%<br>Increase of F&V consumption by a portion/d among 50% of population<br>Stricter tobacco control	£5.11 per invitation<br>£13.28 per participant
E	Targeted with the addition of structural interventions	Coverage: 20% for the most deprived national IMD fifth and 13.8% for all other fifths<br>Uptake: 66% for the most deprived national IMD fifth and 32.3% for all other fifths<br>Prescription: 9.1% (low risk) 25.8% (middle risk) 41.7% (high risk)<br>Risk profile of participants from the most deprived fifth is similar to the risk profile of the population in the most deprived fifth<br>Salt mandatory reformulation<br>SSB tax rate: 20%<br>Increase of F&V consumption by a portion/d among 50% of population<br>Stricter tobacco control	£5.11 per invitation<br>£15.00 per participant

SCENARIO RESULTS

Baseline

This scenario simulates a hypothetical situation in which NHS Health Checks programme has not been implemented. We used this as our baseline scenario, which all other counterfactual scenarios are compared to. The scenario assumes that recent observed trends in population risk factor exposures will continue in the future. It also assumes that case fatality for CHD will improve by 5% (relative) every year, and case fatality for stroke will improve by 2% (relative) every year.

Table E contains $\text{IMPACT}_{\text{NCD}}$ estimates of the baseline scenario for years between 2011 and 2040. Incidence cases and rates include silent and undiagnosed cases and are for the first-ever event. QALYs and disease costs have been discounted by 3.5% annually.

Table E Model estimates for ages 30 to 84 by sex and overall. Results are rounded. Brackets contain 95% uncertainty intervals.

Model estimates	By the year 2011	By the year 2020	By the year 2030	By the year 2040
Population	258,000 (258,000 to 258,000)	283,000 (283,000 to 284,000)	309,000 (309,000 to 310,000)	328,000 (327,000 to 329,000)
Cumulative CHD incidence (Cases)	1,000 (880 to 1,200)	9,700 (9,200 to 10,000)	19,000 (18,000 to 20,000)	29,000 (27,000 to 30,000)
Cumulative stroke incidence (Cases)	610 (500 to 710)	6,000 (5,700 to 6,400)	12,000 (12,000 to 13,000)	19,000 (18,000 to 20,000)
Cumulative CHD mortality (Deaths)	390 (310 to 480)	3,100 (2,900 to 3,300)	4,900 (4,600 to 5,300)	6,100 (5,800 to 6,500)
Cumulative stroke mortality (Deaths)	170 (120 to 220)	1,500 (1,300 to 1,700)	2,700 (2,500 to 2,900)	3,800 (3,600 to 4,100)
Cumulative QALYs (million, discounted)	0.25 (0.24 to 0.26)	2.3 (2.2 to 2.3)	4.0 (3.8 to 4.2)	5.3 (5.1 to 5.5)
Disease costs (£ million, discounted)	130 (94 to 170)	1,100 (800 to 1,400)	1,900 (1,400 to 2,500)	2,400 (1,800 to 3,200)

Scenario A: Current implementation

This scenario models the current local implementation of NHS Health Checks based on the Review of the NHS Health Check Programme in Liverpool report.[40] We used real-life eligibility criteria[*]. We assumed that since 2011 the annual coverage[†] of the intervention has been 13.8% and the uptake[‡] of the intervention has been 32.3%. We modelled the age, sex, fifth of national IMD and risk profile of participants (using QRISK score[41,42]) based on real-life data that the NHS Liverpool CCG kindly shared with us (Table G). The data shows that about 3 out of 4 participants have a low cardiovascular risk (QRISK < 10), about 1 in 5 have a middle cardiovascular risk (QRISK 10 - 20), and only about 1 in 20 have a high cardiovascular risk (QRISK 20+). Subsequently, we assumed a prescription rate[§] of 9.1% for low-risk participants (QRISK < 10), 25.8% for middle-risk participants (QRISK 10 - 20), and 41.7% for high-risk participants (QRISK 20+) again based on real-life data from the NHS Liverpool CCG. The data allowed us to further stratify these estimates by fifth of deprivation (Table H). We assumed an 80% mean persistence[**] with treatment and a mean adherence[††] of approximately 70%, roughly based on evidence from Denmark due to lack of local or national evidence.[33] We assumed that participants will not adopt healthier behaviours in their lifestyle based on the observed low rates of referrals to lifestyle services.[40] Finally, we assumed that the cost of NHS Health Checks from a healthcare perspective is £5.11 per invitation and £13.28 per participant.

We ran this scenario for years 2011 to 2040, and we compared the results with the results from the baseline scenario. We then estimated the number of CVD cases prevented or postponed, the number of CVD deaths prevented or postponed, and the number of non-CVD deaths prevented or postponed, cumulatively between 2011 and 2040. The number of non-CVD deaths prevented or postponed is negative for two reasons. The first reason is that some of the CVD cases that are prevented or postponed, eventually die from other causes at a later age. The second is that statins and antihypertensive medication that may be prescribed after a health check increase the probability of new-onset diabetes. We explicitly model this for antihypertensive medication, but not for statins.

[*] Age 40 to 74 and no previous hypertension, CVD, atrial fibrillation, diabetes mellitus, rheumatoid arthritis or kidney disease.

[†] Eligible population invited divided by eligible population.

[‡] Participants divided by invitees.

[§] Those that have statin or antihypertensive drug issued and recorded anytime following NHS Health Check.

[**] Proportion of those who continue the prescribed medication until the next NHS Health Check.

[††] Proportion of the prescribed dose that is taken by the participant.

Similarly, we estimated the cost-effectiveness of this scenario by cumulatively counting the QALYs gained* as a result of NHS Health Checks between 2011 and 2040, and the net cost (calculated as invitation cost + participation cost + disease cost - disease cost in baseline scenario) over the same period. Then we calculated the ICER, and the NMB assuming a willingness to pay £20,000 per QALY gained (Table F).

Finally, to estimate the equity of NHS Health Checks and their impact on existing absolute and relative socioeconomic inequalities in CVD in Liverpool we used two regression-based metrics inspired by the slope index of inequality;[6] the absolute equity slope index and the relative equity slope index.[1,3] The absolute equity slope index measures the impact of an intervention on absolute inequality and the relative equity slope index considers the pre-existing socioeconomic gradient of disease burden and measures the impact of an intervention on relative inequality. For both metrics, positive values mean the intervention reduces inequality and negative values that the intervention increases inequality. It is worth mentioning that the impact on relative socioeconomic inequalities is meaningful only when the intervention tackles absolute inequalities (i.e. the absolute equity slope index is positive). We present both metrics in Table F. We used fifths of the national IMD as a marker of socioeconomic stratification.

Table F shows that effectiveness, cost-effectiveness, and equity are dynamically evolving with time. Figure D depicts the estimated annual probability of this scenario to be cost-effective (assuming a willingness to pay £20,000 per QALY gained), cost saving, or reduce health inequalities (i.e. decrease both absolute and relative socioeconomic health inequalities) in comparison to the baseline scenario.

In summary, $IMPACT_{NCD}$ suggests that the current implementation of NHS Health Checks is unlikely to be cost-effective before 2040, while it is likely to increase socioeconomic health inequalities further.

* Negative QALYs gained values are caused because antihypertensive medication increases the risk of new onset diabetes mellitus.

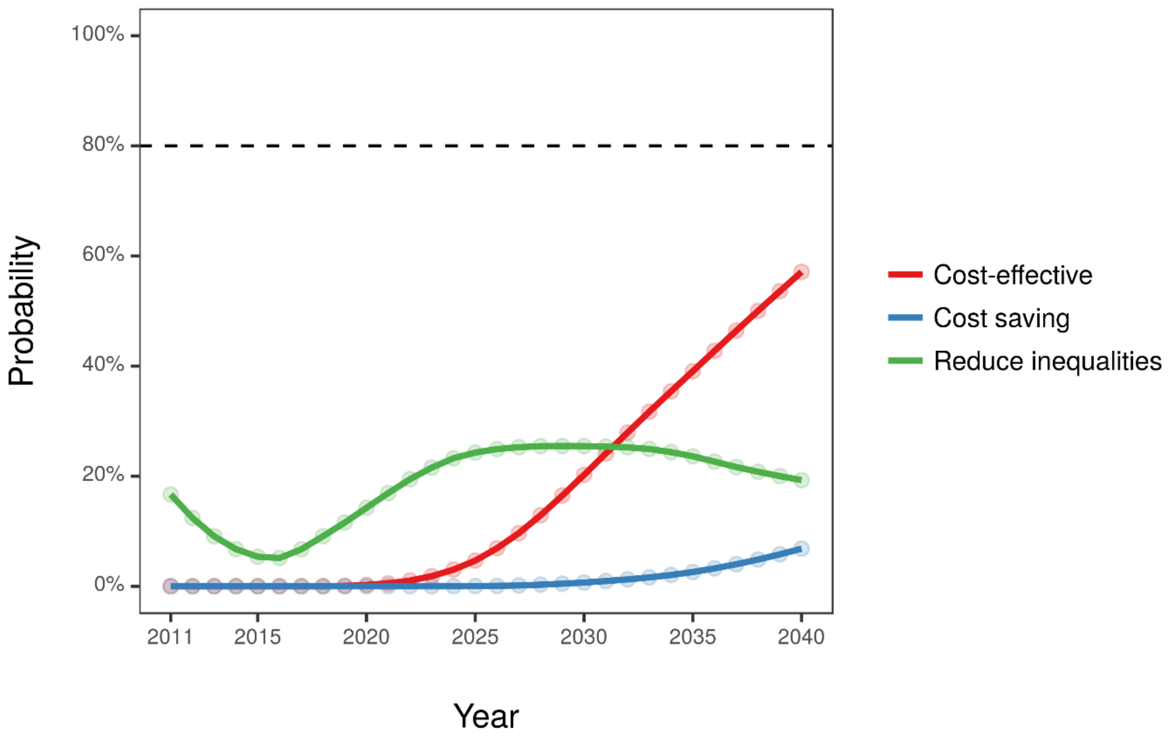

Figure D Annual probability of scenario A to be cost-effective, cost saving, or reduce health inequalities. The horizontal dashed guideline marks the 80% probability, which traditionally used in decision making as a decision rule.

Table F Estimated effectiveness, cost-effectiveness, and equity of current implementation of NHS Health Checks in Liverpool. Ages 30 to 84. Brackets contain 95% uncertainty intervals. Results are rounded to the first 2 significant digits.

**Model estimates**	**By the year 2020**	**By the year 2030**	**By the year 2040**
Cumulative CVD cases prevented or postponed	47<br>(5 to 140)	290<br>(150 to 500)	570<br>(320 to 890)
Cumulative CVD deaths prevented or postponed	0<br>(-5 to 9)	19<br>(0 to 42)	47<br>(14 to 88)
Cumulative deaths from non-CVD causes prevented or postponed	0<br>(-9 to 0)	-14<br>(-33 to 0)	-42<br>(-75 to -14)
Cumulative net QALYs gained (discounted)	-27<br>(-79 to 34)	57<br>(-130 to 310)	220<br>(-110 to 660)
Cumulative invitation cost (discounted £)	910,000<br>(900,000 to 910,000)	1,600,000<br>(1,600,000 to 1,600,000)	2,200,000<br>(2,200,000 to 2,200,000)

Model estimates	By the year 2020	By the year 2030	By the year 2040
Cumulative participation cost (discounted £)	760,000 (750,000 to 770,000)	1,400,000 (1,300,000 to 1,400,000)	1,800,000 (1,800,000 to 1,800,000)
Cumulative net cost (discounted £)	2,900,000 (2,100,000 to 3,700,000)	4,000,000 (1,100,000 to 6,200,000)	3,400,000 (-1,500,000 to 6,900,000)
Cumulative incremental effectiveness ratio (discounted)	-83,000 (-840,000 to 870,000)	21,000 (-650,000 to 730,000)	11,000 (-270,000 to 320,000)
Cumulative net monetary benefit (discounted)	-3,500,000 (-5,000,000 to -1,400,000)	-3,000,000 (-8,300,000 to 5,000,000)	940,000 (-8,600,000 to 14,000,000)
Reduction in absolute socioeconomic health inequalities	-81 (-290 to 150)	150 (-570 to 1,100)	600 (-660 to 2,300)
Reduction in relative socioeconomic health inequalities	13 (-74 to 97)	-24 (-230 to 130)	-76 (-330 to 140)

Table G Age, sex, fifth of national IMD, and risk profile distribution of NHS Health Check participants. Source: NHS Liverpool CCG.

			QRISK RANGE		
**Sex**	**Age range**	**Fifths of national IMD**	**< 10**	**10 to 20**	**20+**
Men	40-49	1 (least deprived)	0.4%	0.0%	0.0%
		2	2.0%	0.0%	0.0%
		3	2.2%	0.0%	0.0%
		4	2.5%	0.1%	0.0%
		5 (most deprived)	8.4%	0.3%	0.0%
	50-59	1 (least deprived)	0.5%	0.1%	0.0%
		2	2.0%	0.3%	0.0%
		3	2.0%	0.5%	0.1%
		4	2.3%	0.6%	0.0%
		5 (most deprived)	5.5%	2.0%	0.4%
	60-69	1 (least deprived)	0.1%	0.3%	0.0%
		2	0.5%	1.0%	0.2%

Sex	Age range	Fifths of national IMD	QRISK RANGE		
			< 10	10 to 20	20+
		3	0.4%	1.1%	0.3%
		4	0.4%	1.1%	0.3%
		5 (most deprived)	0.8%	2.9%	0.9%
	70-74	1 (least deprived)	0.0%	0.1%	0.1%
		2	0.0%	0.3%	0.3%
		3	0.0%	0.3%	0.3%
		4	0.0%	0.2%	0.4%
		5 (most deprived)	0.1%	0.4%	1.1%
Women	40-49	1 (least deprived)	0.5%	0.0%	0.0%
		2	2.3%	0.0%	0.0%
		3	2.6%	0.0%	0.0%
		4	3.0%	0.0%	0.0%
		5 (most deprived)	8.7%	0.1%	0.0%
	50-59	1 (least deprived)	0.6%	0.0%	0.0%
		2	2.9%	0.0%	0.0%
		3	3.1%	0.1%	0.0%
		4	3.5%	0.1%	0.0%
		5 (most deprived)	8.0%	0.6%	0.1%
	60-69	1 (least deprived)	0.4%	0.1%	0.0%
		2	1.8%	0.5%	0.0%
		3	1.7%	0.5%	0.1%
		4	1.6%	0.7%	0.0%
		5 (most deprived)	2.9%	2.0%	0.2%
	70-74	1 (least deprived)	0.0%	0.2%	0.0%
		2	0.1%	0.8%	0.1%
		3	0.1%	0.7%	0.2%
		4	0.1%	0.7%	0.2%
		5 (most deprived)	0.2%	1.2%	0.7%

Table H Prescription rate after an NHS Health Check by fifth of national IMD and risk profile. All first-time prescriptions of a statin or antihypertensive medication issued and recorded anytime following NHS Health Check were included in the numerator. Source: NHS Liverpool CCG.

	**QRISK range**		
**Fifth of national IMD**	**<10**	**10-20**	**20+**
1 (least deprived)	6.0%	23.9%	25.0%
2	7.7%	21.8%	36.8%
3	8.3%	21.8%	40.3%
4	8.9%	24.8%	38.4%
5 (most deprived)	10.1%	28.8%	45.0%
All IMD	9.1%	25.8%	41.7%

Scenario B: Increased annual coverage to 20% and uptake to 66% in the most deprived fifth of national IMD from 2017.

This scenario assumed that in addition to the increased coverage and uptake of the intervention to the most deprived areas, participants from the most deprived fifth of the population share the same risk profile as the eligible population in the most deprived fifth. Therefore, we assume here that the hypothetical recruitment strategy in the most deprived areas manages to attract participants with higher cardiovascular risk profile than in scenario A. This scenario resembles the 'concentrated screening' scenario assumptions in our previous study.[3] For simplicity we will call this scenario, 'targeted'.

Table I and Figure E summarise the results for this scenario. The model suggests that this scenario is more effective, cost-effective, and equitable than the current scenario. In fact, this scenario is likely to be cost-effective and equitable by 2040. The results highlight the importance of the cardiovascular risk profile of the participants on the effectiveness, cost-effectiveness, and equity of NHS Health Checks.

Table I Estimated effectiveness, cost-effectiveness, and equity of the 'targeted' scenario. Ages 30 to 84. Brackets contain 95% uncertainty intervals. Results are rounded to the first 2 significant digits.

Model estimates	By the year 2020	By the year 2030	By the year 2040
Cumulative CVD cases prevented or postponed	42 (5 to 140)	530 (270 to 930)	1,200 (730 to 1,900)
Cumulative CVD deaths prevented or postponed	0 (0 to 9.3)	23 (5 to 65)	84 (33 to 150)
Cumulative deaths from non-CVD causes prevented or postponed	0 (-9 to 0)	-19 (-42 to 0)	-65 (-110 to -33)
Cumulative net QALYs gained (discounted)	-33 (-86 to 33)	85 (-200 to 490)	500 (-82 to 1,300)
Cumulative invitation cost (discounted £)	1,000,000 (990,000 to 1,000,000)	1,900,000 (1,900,000 to 1,900,000)	2,600,000 (2,600,000 to 2,600,000)
Cumulative participation cost (discounted £)	1,100,000 (1,100,000 to 1,100,000)	2,500,000 (2,400,000 to 2,500,000)	3,500,000 (3,500,000 to 3,600,000)
Cumulative net cost (discounted £)	3,500,000 (2,500,000 to 4,200,000)	4,700,000 (-110,000 to 7,900,000)	1,300,000 (-8,600,000 to 7,500,000)
Cumulative incremental effectiveness ratio (discounted)	-85,000 (-760,000 to 840,000)	14,000 (-450,000 to 540,000)	1,500 (-91,000 to 100,000)

Model estimates	By the year 2020	By the year 2030	By the year 2040
Cumulative net monetary benefit (discounted)	-4,200,000 (-5,600,000 to -2,000,000)	-3,000,000 (-11,000,000 to 10,000,000)	8,800,000 (-8,600,000 to 34,000,000)
Reduction in absolute socioeconomic health inequalities	-120 (-340 to 150)	410 (-1,000 to 2,600)	2,900 (-360 to 7,700)
Reduction in relative socioeconomic health inequalities	12 (-76 to 88)	11 (-150 to 200)	120 (-110 to 400)

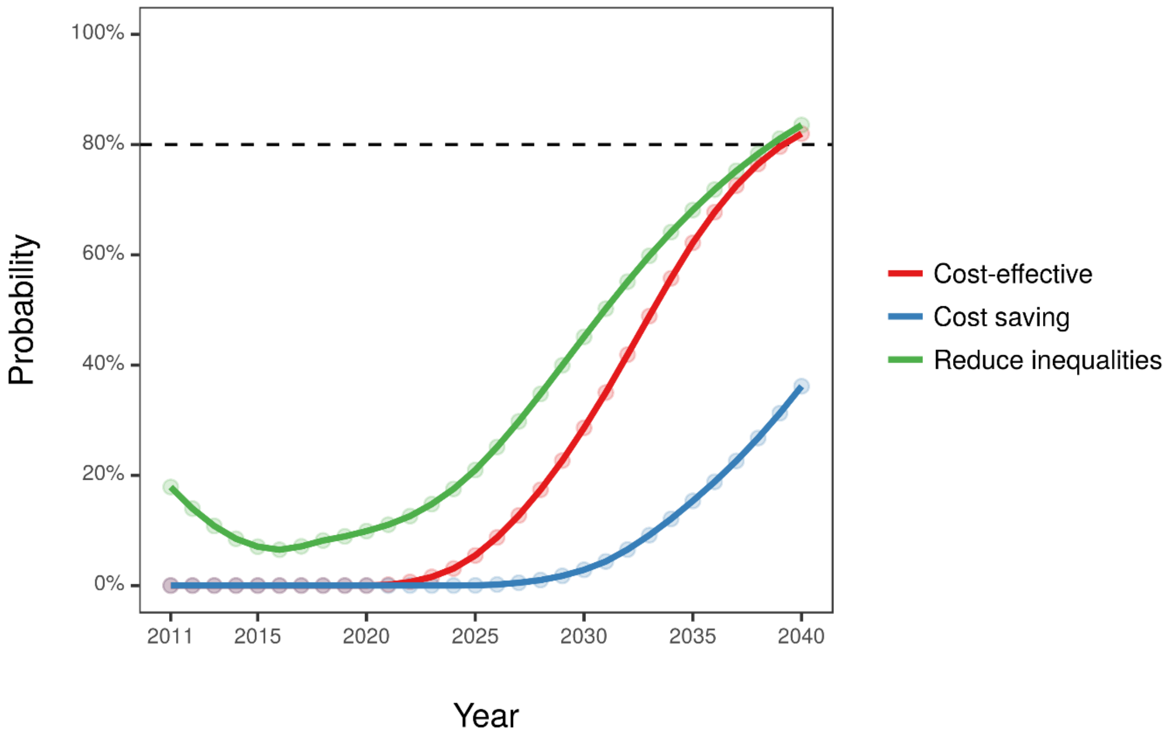

Figure E Annual probability of the 'targeted' scenario to be cost-effective, cost saving, or reduce health inequalities. The horizontal dashed guideline marks the 80% probability, which traditionally used in decision making as a decision rule.

Scenario C: Optimal implementation from 2017

This scenario is similar to the 'current implementation' scenario until 2017. From 2017 onwards, it assumes optimal annual coverage (20%), uptake (66%), prescription rate (9.1% (low risk) 80% (middle risk) 80% (high risk)), and referrals to highly effective lifestyle services. Specifically, it assumes that lifestyle services are highly effective to achieve long-term behavioural change towards healthier lifestyles. Consequently, we assume that 50% of mid- and high-risk participants (QRISK > 10) increase their daily fruit and vegetable consumption by one portion, their physical activity by an active day per week, they decrease their BMI by 1% and those with BMI > 50 kg/m2 will have bariatric surgery and will reduce their BMI to 30 kg/m^2. Finally, this scenario assumes 10% of smokers will achieve smoking cessation for at least 5 years. We did not consider an extra cost from the utilisation of lifestyle services, as these services are already in place. So, we are assuming that individuals who are referred will use existing spare capacity in these services. We assumed an increase in cost per participant to £15.00.

Table J and Figure F summarise the model outputs for this scenario. This is the most effective, and cost-effective scenario that has been presented so far. The model suggested that this scenario is likely to become cost-effective from 2031 onwards and cost saving in 2040. However, it is unlikely to become equitable before 2040.

Table J Estimated effectiveness, cost-effectiveness, and equity of the 'optimal' scenario. Ages 30 to 84. Brackets contain 95% uncertainty intervals. Results are rounded to the first 2 significant digits.

Model estimates	By the year 2020	By the year 2030	By the year 2040
Cumulative CVD cases prevented or postponed	47 (5 to 160)	750 (400 to 1,300)	2,000 (1,400 to 2,900)
Cumulative CVD deaths prevented or postponed	0 (-5 to 9)	33 (5 to 84)	130 (61 to 220)
Cumulative deaths from non-CVD causes prevented or postponed	0 (-5 to 5)	51 (9 to 100)	190 (110 to 270)
Cumulative net QALYs gained (discounted)	-33 (-89 to 42)	310 (-110 to 960)	1,700 (700 to 3,100)
Cumulative invitation cost (discounted £)	1,100,000 (1,100,000 to 1,100,000)	2,100,000 (2,100,000 to 2,100,000)	2,900,000 (2,900,000 to 2,900,000)
Cumulative participation cost (discounted £)	1,300,000 (1,300,000 to 1,300,000)	3,100,000 (3,100,000 to 3,100,000)	4,500,000 (4,500,000 to 4,500,000)
Cumulative net cost (discounted £)	3,700,000 (2,800,000 to 4,600,000)	3,900,000 (-2,800,000 to 8,200,000)	-4,200,000 (-18,000,000 to 4,300,000)

Model estimates	By the year 2020	By the year 2030	By the year 2040
Cumulative incremental effectiveness ratio (discounted)	-91,000 (-970,000 to 940,000)	9,700 (-170,000 to 190,000)	-2,400 (-6,500 to 5,700)
Cumulative net monetary benefit (discounted)	-4,500,000 (-6,000,000 to -2,000,000)	2,500,000 (-10,000,000 to 22,000,000)	38,000,000 (10,000,000 to 79,000,000)
Reduction in absolute socioeconomic health inequalities	-89 (-300 to 180)	1,300 (-340 to 3,900)	7,200 (3,100 to 13,000)
Reduction in relative socioeconomic health inequalities	23 (-67 to 120)	-2.1 (-270 to 210)	-50 (-440 to 270)

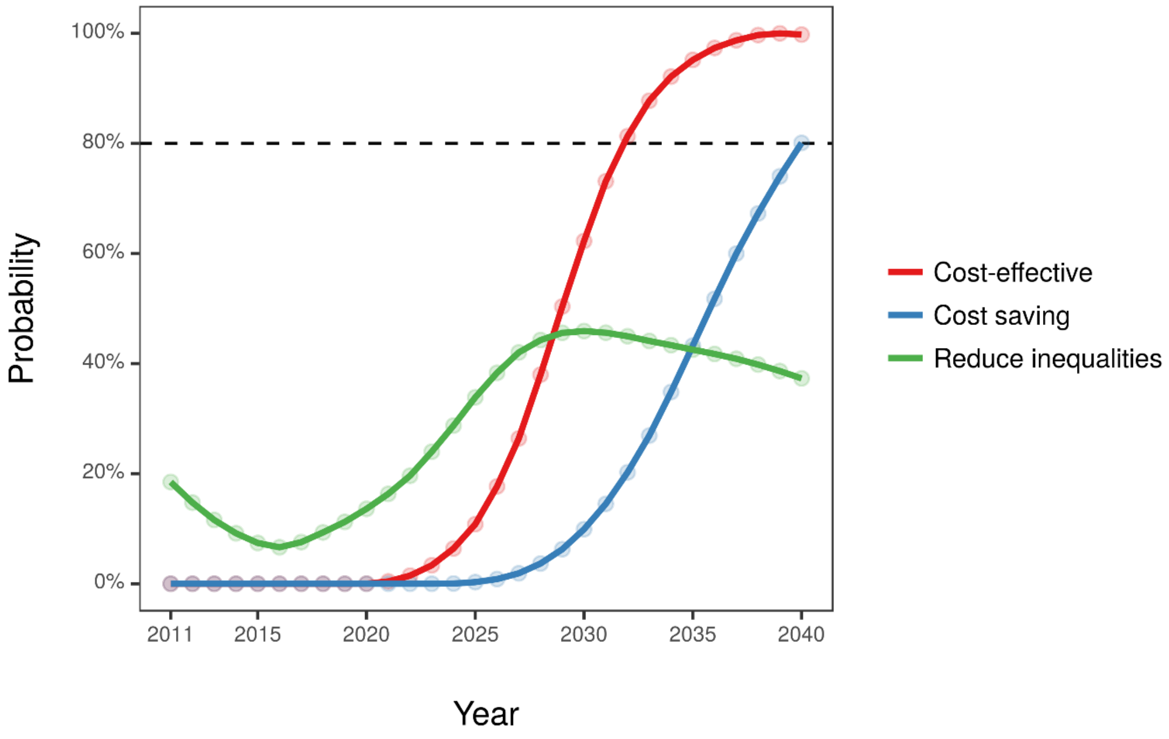

Figure F Annual probability of the ‘optimal’ scenario to be cost-effective, cost saving, or reduce health inequalities. The horizontal dashed guideline marks the 80% probability, which traditionally used in decision making as a decision rule.

Scenario D: Current implementation + structural interventions

This is the combination of the 'current scenario with the following four structural interventions;

1. Mandatory Salt Reformulation

From 2017 onwards, it assumes that mandatory salt reformulation of processed foods can reduce the mean salt daily consumption down to 6g (the national target) within five years. This would result in a further decrease in systolic blood pressure and fewer cases of CVD. We modelled the absolute effect of salt reformulation on systolic blood pressure for the population of Liverpool by year, age, sex, and QIMD based on previous research by Kypridemos et al.[2] The mean decrease in systolic blood pressure was estimated to be approximately 0.3 mmHg.

2. 20% Sugar-Sweetened Beverages Tax

From 2017 onwards, it assumes that a 20% SSB tax is implemented that would reduce BMI. We modelled the tax effect on Liverpool population based on findings from Briggs *et al*.[34 Table 8] We assumed the lowest income third is QIMD 5 and highest income third is QIMD 1. The mean decrease in BMI in this scenario was approximately 0.05 kg/m^2.

3. Increase of fruit and vegetable consumption by a portion/day among 50% of population

From 2017 onwards, it assumes that the daily fruit and vegetable (F&V) consumption is increased by a portion (80g) for 50% of the population in Liverpool. We modelled this increase of F&V consumption roughly based on studies from Bartlett et al. and Nnoaham et al.[43,44]

4. Stricter Tobacco Control

From 2017 onwards, it assumes that a proportional decrease in smoking prevalence by age, sex, and QIMD is observed in the population because of stricter anti-tobacco legislation. We based this scenario on a study from Allen K, et al. that modelled the potential effect of maximising the Tobacco Control Scale in the UK.[35] The mean effect of this policy was approximately a 5% absolute decrease in smoking prevalence for ages 30 to 84 (from about 30% to about 25%).

We did not consider any implementation cost for the structural policies above because we adopt a healthcare perspective in our cost-effectiveness analysis.

Table K and Figure G summarise the model outputs for this scenario. $IMPACT_{NCD}$ suggested that this scenario is likely to become cost-effective by 2023, cost saving by 2025, and equitable by 2022.

Table K Estimated effectiveness, cost-effectiveness, and equity of the ‘current + structural’ scenario. Ages 30 to 84. Brackets contain 95% uncertainty intervals. Results are rounded to the first 2 significant digits.

**Model estimates**	**By the year 2020**	**By the year 2030**	**By the year 2040**
Cumulative CVD cases prevented or postponed	84 (14 to 420)	1,600 (1,000 to 2,300)	3,300 (2,400 to 4,200)
Cumulative CVD deaths prevented or postponed	0 (0 to 19)	70 (23 to 140)	210 (120 to 330)
Cumulative deaths from non-CVD causes prevented or postponed	0 (-5 to 130)	540 (320 to 750)	1,000 (680 to 1,300)
Cumulative net QALYs gained (discounted)	6 (-47 to 290)	2,400 (1,100 to 4,300)	7,000 (4,600 to 10,000)
Cumulative invitation cost (discounted £)	890,000 (890,000 to 900,000)	1,600,000 (1,600,000 to 1,600,000)	2,100,000 (2,100,000 to 2,100,000)
Cumulative participation cost (discounted £)	750,000 (740,000 to 760,000)	1,300,000 (1,300,000 to 1,300,000)	1,800,000 (1,800,000 to 1,800,000)
Cumulative net cost (discounted £)	1,500,000 (-1,500,000 to 2,600,000)	-13,000,000 (-28,000,000 to -3,700,000)	-35,000,000 (-60,000,000 to -19,000,000)
Cumulative incremental effectiveness ratio (discounted)	-2,600 (-750,000 to 740,000)	-5,200 (-8,400 to -2,600)	-5,100 (-7,400 to -3,200)
Cumulative net monetary benefit (discounted)	-1,400,000 (-3,300,000 to 7,000,000)	62,000,000 (27,000,000 to 110,000,000)	180,000,000 (120,000,000 to 250,000,000)
Reduction in absolute socioeconomic health inequalities	67 (-130 to 1,400)	13,000 (5,800 to 22,000)	37,000 (24,000 to 52,000)
Reduction in relative socioeconomic health inequalities	24 (-79 to 140)	550 (160 to 1,100)	1,200 (630 to 1,900)

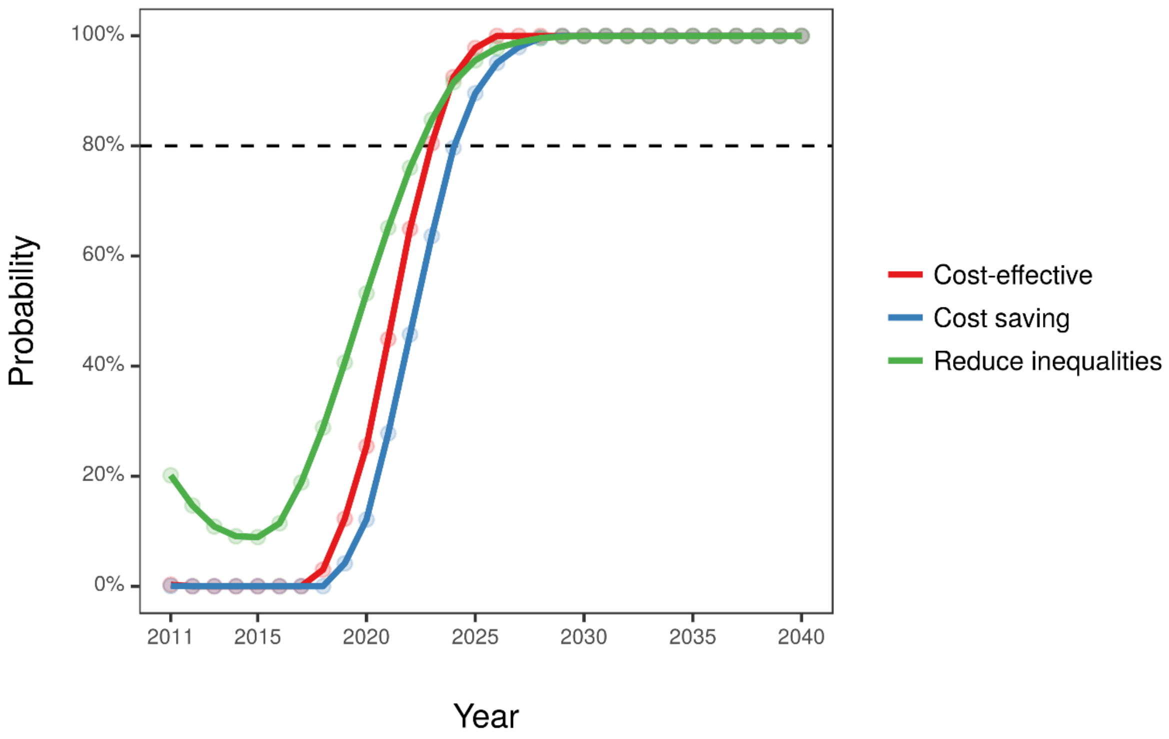

Figure G Annual probability of the 'current + structural' scenario to be cost-effective, cost saving, or reduce health inequalities. The horizontal dashed guideline marks the 80% probability, which traditionally used in decision making as a decision rule.

Scenario E: Targeted implementation + all structural interventions

This is the combination of all the structural interventions outlined previously in scenario D with the 'targeted' scenario (scenario B).

Table L and Figure H summarise the model outputs for this scenario. $IMPACT_{NCD}$ suggested that this scenario is likely to become cost-effective by 2024, cost saving by 2026, and equitable by 2024.

Table L Estimated effectiveness, cost-effectiveness, and equity of the 'targeted + structural' scenario. Ages 30 to 84. Brackets contain 95% uncertainty intervals. Results are rounded to the first 2 significant digits.

Model estimates	By the year 2020	By the year 2030	By the year 2040
Cumulative CVD cases prevented or postponed	84 (14 to 450)	1,800 (1,100 to 2,700)	3,800 (2,900 to 5,000)
Cumulative CVD deaths prevented or postponed	0 (0 to 19)	79 (28 to 150)	240 (140 to 380)
Cumulative deaths from non-CVD causes prevented or postponed	0 (5 to 120)	540 (330 to 750)	1,000 (650 to 1,300)
Cumulative net QALYs gained (discounted)	1 (-57 to 290)	2,400 (1,000 to 4,500)	7,200 (4,700 to 10,000)
Cumulative invitation cost (discounted £)	1,000,000 (1,000,000 to 1,000,000)	1,900,000 (1,900,000 to 1,900,000)	2,600,000 (2,600,000 to 2,700,000)
Cumulative participation cost (discounted £)	1,100,000 (1,100,000 to 1,100,000)	2,500,000 (2,500,000 to 2,500,000)	3,500,000 (3,500,000 to 3,600,000)
Cumulative net cost (discounted £)	2,100,000 (-740,000 to 3,200,000)	-11,000,000 (-27,000,000 to -1,700,000)	-35,000,000 (-63,000,000 to -18,000,000)
Cumulative incremental effectiveness ratio (discounted)	-6,400 (-820,000 to 930,000)	-4,600 (-7,700 to -1,400)	-5,000 (-7,400 to -3,100)
Cumulative net monetary benefit (discounted)	-2,100,000 (-4,100,000 to 6,400,000)	60,000,000 (23,000,000 to 110,000,000)	180,000,000 (120,000,000 to 270,000,000)

Model estimates	By the year 2020	By the year 2030	By the year 2040
Reduction in absolute socioeconomic health inequalities	14<br>(-200 to 1,400)	13,000<br>(5,300 to 23,000)	38,000<br>(25,000 to 55,000)
Reduction in relative socioeconomic health inequalities	11<br>(-88 to 120)	550<br>(130 to 1,200)	1,300<br>(670 to 2,000)

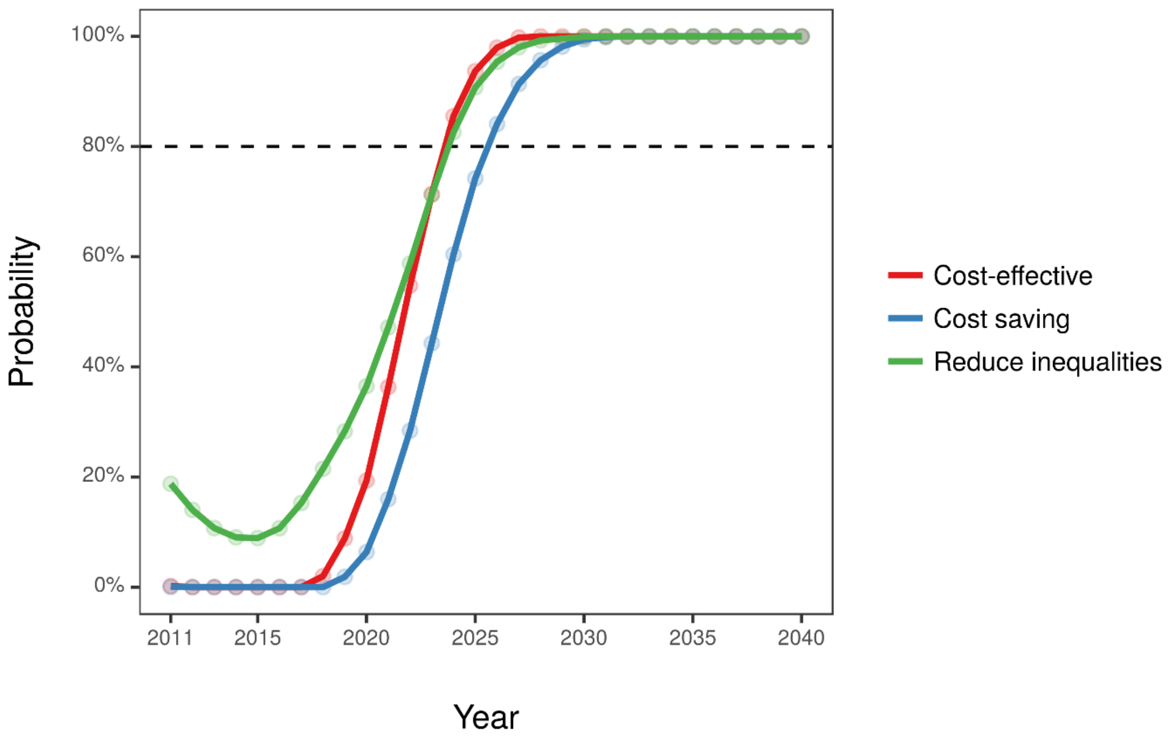

Figure H Annual probability of the 'targeted + structural' scenario to be cost-effective, cost saving, or reduce health inequalities. The horizontal dashed guideline marks the 80% probability, which traditionally used in decision making as a decision rule.

Between scenario comparisons (probability of superiority)

In the 'uncertainty' section above (p9), we wrote that overlapping UIs is not evidence against statistical significance because the scenarios are share common sources of uncertainty. In the following tables we summarise the probability of superiority of each scenario to be more cost-effective in comparison to all other modelled scenarios. We calculate the probability of superiority for a scenario to be more cost-effective than a counterfactual as the proportion of Monte Carlo simulations in which the estimated NMB for this scenario was higher than the counterfactual. We did the same for absolute and relative inequalities.

Table M Between scenario comparisons. Probability of each scenario to be more cost-effective than counterfactual scenarios by 2030.

	Current (A)	*Current + Targeted (B)*	*Optimal (C)*	*Current + Structural (D)*	*Current + Targeted + Structural (E)*
Probability of Current (A) scenario being superior to...	0	0.5165	0.132	0	0
Probability of Current + Targeted (B) scenario being superior to...	0.4835	0	0.048	0	0
Probability of Optimal (C) scenario being superior to...	0.868	0.952	0	0	0
Probability of Current + Structural (D) scenario being superior to...	1	1	1	0	0.623
Probability of Current + Targeted + Structural (E) scenario being superior to...	1	1	1	0.377	0

Table N Between scenario comparisons. Probability of each scenario to be more cost-effective than counterfactual scenarios by 2040.

	Current (A)	*Current + Targeted (B)*	*Optimal (C)*	*Current + Structural (D)*	*Current + Targeted + Structural (E)*
Probability of Current (A) scenario being superior to...	0	0.0805	0	0	0
Probability of Current + Targeted (B) scenario being superior to...	0.9195	0	0	0	0
Probability of Optimal (C) scenario being superior to...	1	1	0	0	0
Probability of Current + Structural (D) scenario being superior to...	1	1	1	0	0.3235
Probability of Current + Targeted + Structural (E) scenario being superior to...	1	1	1	0.6765	0

Table O Between scenario comparisons. Probability of each scenario to be more equitable in terms of absolute inequalities than counterfactual scenarios by 2030.

	Current (A)	*Current + Targeted (B)*	*Optimal (C)*	*Current + Structural (D)*	*Current + Targeted + Structural (E)*
Probability of Current (A) scenario being superior to...	0	0.3505	0.0435	0	0
Probability of Current + Targeted (B) scenario being superior to...	0.6495	0	0.0435	0	0
Probability of Optimal (C) scenario being superior to...	0.9565	0.9565	0	0	0
Probability of Current + Structural (D) scenario being superior to...	1	1	1	0	0.5135
Probability of Current + Targeted + Structural (E) scenario being superior to...	1	1	1	0.4865	0

Table P Between scenario comparisons. Probability of each scenario to be more equitable in terms of absolute inequalities than counterfactual scenarios by 2040.

	Current (A)	*Current + Targeted (B)*	*Optimal (C)*	*Current + Structural (D)*	*Current + Targeted + Structural (E)*
Probability of Current (A) scenario being superior to...	0	0.0415	0	0	0
Probability of Current + Targeted (B) scenario being superior to...	0.9585	0	0	0	0
Probability of Optimal (C) scenario being superior to...	1	1	0	0	0
Probability of Current + Structural (D) scenario being superior to...	1	1	1	0	0.299
Probability of Current + Targeted + Structural (E) scenario being superior to...	1	1	1	0.701	0

Table Q Between scenario comparisons. Probability of each scenario to be more equitable in terms of both absolute and relative inequalities than counterfactual scenarios by 2030.

	Current (A)	*Current + Targeted (B)*	*Optimal (C)*	*Current + Structural (D)*	*Current + Targeted + Structural (E)*
Probability of Current (A) scenario being superior to...	0	0.238	0.031	0	0
Probability of Current + Targeted (B) scenario being superior to...	0.5055	0	0.0415	0	0
Probability of Optimal (C) scenario being superior to...	0.5565	0.4555	0	0	0
Probability of Current + Structural (D) scenario being superior to...	0.998	0.9975	0.9985	0	0.396
Probability of Current + Targeted + Structural (E) scenario being superior to...	0.996	0.996	0.994	0.361	0

Table R Between scenario comparisons. Probability of each scenario to be more equitable in terms of both absolute and relative inequalities than counterfactual scenarios by 2040.

	Current (A)	*Current + Targeted (B)*	*Optimal (C)*	*Current + Structural (D)*	*Current + Targeted + Structural (E)*
Probability of Current (A) scenario being superior to...	0	0.0255	0	0	0
Probability of Current + Targeted (B) scenario being superior to...	0.88	0	0	0	0
Probability of Optimal (C) scenario being superior to...	0.5485	0.1965	0	0	0
Probability of Current + Structural (D) scenario being superior to...	1	1	1	0	0.217
Probability of Current + Targeted + Structural (E) scenario being superior to...	1	1	1	0.565	0

CVD BURDEN PROJECTIONS FOR LIVERPOOL

$IMPACT_{NCD}$ estimates CHD and stroke incidence, prevalence, and mortality rates by year for the scenarios. In the following tables, we present these for the 'current' scenario (scenario 2). These modelled estimates are not forecasts, but they may be useful in future modelling exercises. They can also provide a better understanding of the impact of our modelling assumptions.

Table S $IMPACT_{NCD}$ estimates of CVD burden in Liverpool. Ages 30 to 84. Brackets contain 95% uncertainty intervals. Results are rounded to the first 2 significant digits (3 for population estimates).

**Model estimates**	**Year 2020**	**Year 2030**	**Year 2040**
Population	283,000 (283,000 to 284,000)	309,000 (309,000 to 310,000)	328,000 (327,000 to 329,000)
CHD incidence rate per 100,000 population	330 (280 to 370)	300 (260 to 350)	290 (250 to 340)
CHD prevalence rate per 100,000 population	5,500 (5,300 to 5,600)	5,000 (4,800 to 5,200)	4,700 (4,400 to 5,000)
CHD mortality rate per 100,000	84 (62 to 110)	48 (33 to 66)	28 (17 to 43)
Stroke incidence rate per 100,000 population	210 (180 to 250)	210 (170 to 250)	210 (170 to 240)
Stroke prevalence rate per 100,000 population	3,300 (3,100 to 3,400)	3,100 (3,000 to 3,300)	3,000 (2,800 to 3,200)
Stroke mortality rate per 100,000	46 (31 to 66)	38 (24 to 54)	31 (20 to 46)

Table T IMPACT$_{NCD}$ estimates of CVD burden in Liverpool by sex. Ages 30 to 84. Brackets contain 95% uncertainty intervals. Results are rounded to the first 2 significant digits (3 for population estimates).

Sex	Model estimates	Year 2020	Year 2030	Year 2040
Men	Population	141,000 (141,000 to 142,000)	157,000 (156,000 to 157,000)	168,000 (168,000 to 169,000)
	CHD incidence rate per 100,000 population	440 (370 to 520)	420 (350 to 500)	420 (350 to 500)
	CHD prevalence rate per 100,000 population	6,700 (6,500 to 7,000)	6,400 (6,100 to 6,700)	6,200 (5,800 to 6,600)
	CHD mortality rate per 100,000	120 (82 to 160)	68 (42 to 98)	41 (22 to 64)
	Stroke incidence rate per 100,000 population	260 (200 to 310)	260 (210 to 320)	270 (210 to 330)
	Stroke prevalence rate per 100,000 population	3,800 (3,600 to 4,100)	3,700 (3,500 to 3,900)	3,600 (3,400 to 3,900)
	Stroke mortality rate per 100,000	49 (26 to 76)	42 (21 to 66)	33 (17 to 56)
Women	Population	142,000 (142,000 to 142,000)	153,000 (152,000 to 153,000)	160,000 (159,000 to 160,000)
	CHD incidence rate per 100,000 population	210 (160 to 270)	180 (130 to 230)	160 (120 to 210)
	CHD prevalence rate per 100,000 population	4,200 (4,000 to 4,400)	3,600 (3,400 to 3,800)	3,100 (2,800 to 3,400)
	CHD mortality rate per 100,000	52 (29 to 76)	27 (12 to 46)	15 (2.9 to 29)
	Stroke incidence rate per 100,000 population	170 (120 to 220)	160 (110 to 200)	140 (100 to 180)
	Stroke prevalence rate per 100,000 population	2,700 (2,500 to 2,900)	2,500 (2,400 to 2,700)	2,300 (2,100 to 2,500)
	Stroke mortality rate per 100,000	43 (23 to 72)	34 (15 to 58)	29 (12 to 49)

Table U $IMPACT_{NCD}$ estimates of CVD burden in Liverpool by age group. Brackets contain 95% uncertainty intervals. Results are rounded to the first 2 significant digits (3 for population estimates).

Age group	Model estimates	Year 2020	Year 2030	Year 2040
30 to 49	Population	132,000 (131,000 to 132,000)	144,000 (144,000 to 144,000)	144,000 (143,000 to 144,000)
	CHD incidence rate per 100,000 population	110 (78 to 160)	100 (68 to 140)	91 (58 to 130)
	CHD prevalence rate per 100,000 population	990 (870 to 1,100)	950 (840 to 1,100)	850 (720 to 980)
	CHD mortality rate per 100,000	7.1 (0 to 21)	3.2 (0 to 13)	3.2 (0 to 9.7)
	Stroke incidence rate per 100,000 population	64 (35 to 96)	58 (32 to 91)	52 (26 to 78)
	Stroke prevalence rate per 100,000 population	720 (620 to 820)	670 (570 to 770)	550 (460 to 640)
	Stroke mortality rate per 100,000	3.5 (0 to 14)	3.2 (0 to 9.7)	3.2 (0 to 9.7)
50 to 69	Population	109,000 (109,000 to 109,000)	112,000 (112,000 to 113,000)	122,000 (122,000 to 123,000)
	CHD incidence rate per 100,000 population	390 (320 to 470)	340 (270 to 430)	310 (240 to 380)
	CHD prevalence rate per 100,000 population	6,600 (6,300 to 7,000)	5,600 (5,300 to 6,000)	4,700 (4,400 to 5,100)
	CHD mortality rate per 100,000	68 (38 to 110)	37 (17 to 62)	19 (3.8 to 38)
	Stroke incidence rate per 100,000 population	250 (190 to 320)	240 (180 to 310)	220 (160 to 280)
	Stroke prevalence rate per 100,000 population	3,800 (3,500 to 4,000)	3,400 (3,200 to 3,700)	3,000 (2,700 to 3,300)
	Stroke mortality rate per 100,000	30 (8.5 to 51)	21 (4.2 to 41)	15 (3.8 to 31)
70 to 84	Population	42,600 (42,200 to 43,000)	53,300 (52,800 to 53,800)	62,100 (61,500 to 62,600)
	CHD incidence rate per 100,000 population	810 (630 to 1,000)	760 (600 to 930)	730 (580 to 900)
	CHD prevalence rate per 100,000 population	16,000 (15,000 to 17,000)	15,000 (14,000 to 15,000)	14,000 (13,000 to 14,000)

Age group	Model estimates	Year 2020	Year 2030	Year 2040
	CHD mortality rate per 100,000	350 (240 to 480)	190 (120 to 280)	110 (60 to 170)
	Stroke incidence rate per 100,000 population	560 (420 to 720)	550 (410 to 690)	540 (410 to 680)
	Stroke prevalence rate per 100,000 population	9,900 (9,300 to 11,000)	9,100 (8,500 to 9,700)	8,700 (8,100 to 9,300)
	Stroke mortality rate per 100,000	220 (130 to 330)	170 (96 to 250)	130 (75 to 200)

Table V IMPACT$_{NCD}$ estimates of CVD burden in Liverpool by QIMD. Ages 30 to 84. QIMD 1 point estimates are unreliable due to small population size. Brackets contain 95% uncertainty intervals. Results are rounded to the first 2 significant digits (3 for population estimates).

QIMD	Model estimates	Year 2020	Year 2030	Year 2040
1 (least deprived	Population	1,540 (1,500 to 1,570)	1,300 (1,260 to 1,350)	992 (945 to 1,030)
	CHD incidence rate per 100,000 population	310 (0 to 1,200)	360 (0 to 1,400)	470 (0 to 1,800)
	CHD prevalence rate per 100,000 population	5,600 (3,300 to 8,100)	6,800 (4,200 to 9,900)	7,300 (4,200 to 11,000)
	CHD mortality rate per 100,000	0 (0 to 600)	0 (0 to 370)	0 (0 to 480)
	Stroke incidence rate per 100,000 population	300 (0 to 900)	0 (0 to 1,100)	0 (0 to 1,400)
	Stroke prevalence rate per 100,000 population	2,700 (1,200 to 4,500)	3,200 (1,400 to 5,500)	3,500 (1,400 to 6,300)
	Stroke mortality rate per 100,000	0 (0 to 310)	0 (0 to 370)	0 (0 to 480)
2	Population	20,300 (20,200 to 20,500)	20,000 (19,800 to 20,200)	18,700 (18,500 to 18,900)
	CHD incidence rate per 100,000 population	270 (140 to 440)	260 (120 to 420)	220 (99 to 400)
	CHD prevalence rate per 100,000 population	5,200 (4,600 to 5,900)	5,000 (4,300 to 5,700)	4,400 (3,700 to 5,100)
	CHD mortality rate per 100,000	68 (0 to 140)	46 (0 to 120)	25 (0 to 75)

QIMD	Model estimates	Year 2020	Year 2030	Year 2040
	Stroke incidence rate per 100,000 population	160 (68 to 280)	160 (70 to 320)	170 (50 to 300)
	Stroke prevalence rate per 100,000 population	2,100 (1,700 to 2,500)	2,200 (1,800 to 2,700)	2,100 (1,700 to 2,600)
	Stroke mortality rate per 100,000	46 (0 to 110)	46 (0 to 120)	25 (0 to 100)
3	Population	31,600 (31,500 to 31,800)	33,100 (32,900 to 33,300)	33,300 (33,100 to 33,600)
	CHD incidence rate per 100,000 population	310 (180 to 440)	310 (180 to 450)	310 (190 to 430)
	CHD prevalence rate per 100,000 population	4,800 (4,400 to 5,400)	4,700 (4,200 to 5,200)	4,500 (3,900 to 5,000)
	CHD mortality rate per 100,000	59 (15 to 130)	42 (0 to 98)	28 (0 to 70)
	Stroke incidence rate per 100,000 population	220 (120 to 340)	230 (130 to 350)	220 (130 to 350)
	Stroke prevalence rate per 100,000 population	2,900 (2,500 to 3,300)	3,000 (2,600 to 3,400)	3,000 (2,600 to 3,400)
	Stroke mortality rate per 100,000	44 (0 to 100)	28 (0 to 85)	28 (0 to 84)
4	Population	44,900 (44,700 to 45,100)	50,600 (50,400 to 50,900)	54,600 (54,300 to 54,900)
	CHD incidence rate per 100,000 population	300 (200 to 420)	280 (180 to 370)	270 (180 to 370)
	CHD prevalence rate per 100,000 population	4,800 (4,400 to 5,300)	4,500 (4,100 to 5,000)	4,200 (3,800 to 4,700)
	CHD mortality rate per 100,000	72 (21 to 120)	46 (9.2 to 92)	26 (0 to 60)
	Stroke incidence rate per 100,000 population	210 (120 to 310)	210 (130 to 300)	210 (130 to 310)
	Stroke prevalence rate per 100,000 population	2,900 (2,600 to 3,200)	2,900 (2,500 to 3,200)	2,800 (2,400 to 3,100)
	Stroke mortality rate per 100,000	41 (10 to 83)	37 (9.2 to 74)	26 (0 to 60)
5 (most deprived)	Population	185,000 (184,000 to 185,000)	204,000 (204,000 to 205,000)	220,000 (220,000 to 221,000)
	CHD incidence rate per 100,000 population	340 (280 to 400)	310 (260 to 370)	300 (250 to 360)
	CHD prevalence rate per 100,000 population	5,700 (5,500 to 6,000)	5,200 (4,900 to 5,400)	4,800 (4,600 to 5,200)

QIMD	Model estimates	Year 2020	Year 2030	Year 2040
	CHD mortality rate per 100,000	93 (65 to 130)	52 (34 to 75)	30 (17 to 47)
	Stroke incidence rate per 100,000 population	220 (170 to 260)	210 (160 to 260)	200 (160 to 250)
	Stroke prevalence rate per 100,000 population	3,600 (3,400 to 3,800)	3,300 (3,100 to 3,500)	3,100 (2,900 to 3,400)
	Stroke mortality rate per 100,000	50 (30 to 75)	39 (23 to 59)	32 (17 to 49)

References

1. Kypridemos C. Modelling the effectiveness and equity of primary prevention policies in England: a stochastic dynamic microsimulation for the joint prevention of non communicable diseases [Internet]. PhD Thesis, University of Liverpool. 2016. Available: https://elements.liverpool.ac.uk/repository.html?pub=0&com=get-file&rfurl=http%3A%2F%2Flivrepository.liverpool.ac.uk%2Frt4eprints%2Ffile%2F87606%2F201001644_Oct2016.pdf

2. Kypridemos C, Guzman-Castillo M, Hyseni L, Hickey GL, Bandosz P, Buchan I, et al. Estimated reductions in cardiovascular and gastric cancer disease burden through salt policies in England: an IMPACTNCD microsimulation study. BMJ Open. 2017;7: e013791. doi:10/f9mjnf

3. Kypridemos C, Allen K, Hickey GL, Guzman-Castillo M, Bandosz P, Buchan I, et al. Cardiovascular screening to reduce the burden from cardiovascular disease: microsimulation study to quantify policy options. BMJ. 2016;353: i2793. doi:10/gc5p6d

4. Office for National Statistics. Subnational Population Projections for Local Authorities in England: Table 2 [Internet]. 2016 [cited 5 Mar 2018]. Available: https://www.ons.gov.uk/peoplepopulationandcommunity/populationandmigration/populationprojections/datasets/localauthoritiesinenglandtable2

5. Barendregt JJ, van Oortmarssen GJ, Vos T, Murray CJ. A generic model for the assessment of disease epidemiology: the computational basis of DisMod II. Popul Health Metr. 2003;1: 4. doi:10/bgr3m7

6. Mackenbach JP, Kunst AE. Measuring the magnitude of socio-economic inequalities in health: An overview of available measures illustrated with two examples from Europe. Soc Sci Med. 1997;44: 757–771. doi:10/fkcz68

7. Smolina K, Wright FL, Rayner M, Goldacre MJ. Determinants of the decline in mortality from acute myocardial infarction in England between 2002 and 2010: linked national database study. BMJ. 2012;344: d8059. doi:10/fxxzwt

8. Koerkamp BG, Stijnen T, Weinstein MC, Hunink MGM. The combined analysis of uncertainty and patient heterogeneity in medical decision models. Med Decis Making. 2011;31: 650–661. doi:10/cdf2wf

9. Briggs AH, Weinstein MC, Fenwick EAL, Karnon J, Sculpher MJ, Paltiel AD. Model parameter estimation and uncertainty: a report of the ISPOR-SMDM modeling good research practices Task Force-6. Value Health. 2012;15: 835–842. doi:10/f39s8w

10. Jones AM, Lomas J, Rice N. Applying beta-type size distributions to healthcare cost regressions. J Appl Econom. 2014;29: 649–670. doi:10/f5ztkb

11. Berk ML, Monheit AC. The concentration of health care expenditures, revisited. Health Aff Proj Hope. 2001;20: 9–18.

12. Ezzati M, Henley SJ, Thun MJ, Lopez AD. Role of smoking in global and regional cardiovascular mortality. Circulation. 2005;112: 489–497. doi:10/cnmhxf

13. Huxley RR, Woodward M. Cigarette smoking as a risk factor for coronary heart disease in women compared with men: a systematic review and meta-analysis of prospective cohort studies. The Lancet. 2011;378: 1297–1305. doi:10/fq2hx7

14. He J, Vupputuri S, Allen K, Prerost MR, Hughes J, Whelton PK. Passive smoking and the risk of coronary heart disease — a meta-analysis of epidemiologic studies. N Engl J Med. 1999;340: 920–926. doi:10/dhrrzh

15. Prospective Studies Collaboration. Age-specific relevance of usual blood pressure to vascular mortality: a meta-analysis of individual data for one million adults in 61 prospective studies. The Lancet. 2002;360: 1903–1913. doi:10/bzsqjj

16. Prospective Studies Collaboration. Blood cholesterol and vascular mortality by age, sex, and blood pressure: a meta-analysis of individual data from 61 prospective studies with 55 000 vascular deaths. The Lancet. 2007;370: 1829–1839. doi:10/cqhk2v

17. The Emerging Risk Factors Collaboration. Separate and combined associations of body-mass index and abdominal adiposity with cardiovascular disease: collaborative analysis of 58 prospective studies. The Lancet. 2011;377: 1085–1095. doi:10/c69p77

18. The Emerging Risk Factors Collaboration. Diabetes mellitus, fasting blood glucose concentration, and risk of vascular disease: a collaborative meta-analysis of 102 prospective studies. The Lancet. 2010;375: 2215–2222. doi:10/b2qgmq

19. Bull FC, Armstrong TP, Dixon T, Ham S, Neiman A, Pratt M. Comparative quantification of health risks. Chapter 10: physical inactivity [Internet]. Geneva: World Health Organisation; 2004. Available: http://www.who.int/publications/cra/en/

20. Dauchet L, Amouyel P, Hercberg S, Dallongeville J. Fruit and vegetable consumption and risk of coronary heart disease: a meta-analysis of cohort studies. J Nutr. 2006;136: 2588–2593.

21. Oono IP, Mackay DF, Pell JP. Meta-analysis of the association between secondhand smoke exposure and stroke. J Public Health. 2011;33: 496–502. doi:10/b3vd52

22. Dauchet L, Amouyel P, Dallongeville J. Fruit and vegetable consumption and risk of stroke A meta-analysis of cohort studies. Neurology. 2005;65: 1193–1197. doi:10/cvr2gt

23. Kind P, Hardman G, Macran S. UK population norms for EQ-5D [Internet]. Centre for Health Economics, University of York; 1999. Report No.: 172chedp. Available: https://ideas.repec.org/p/chy/respap/172chedp.html

24. Mistry H, Morris S, Dyer M, Kotseva K, Wood D, Buxton M, et al. Cost-effectiveness of a European preventive cardiology programme in primary care: a Markov modelling approach. BMJ Open. 2012;2. doi:10/gb3tpb

25. Tengs TO, Lin TH. A meta-analysis of quality-of-life estimates for stroke. PharmacoEconomics. 2003;21: 191–200.

26. Clarke P, Gray A, Holman R. Estimating utility values for health states of type 2 diabetic patients using the EQ-5D (UKPDS 62). Med Decis Mak Int J Soc Med Decis Mak. 2002;22: 340–349. doi:10/b7t9w9

27. National Institute for Health & Care Excellence. NG 28; Type 2 diabetes in adults: management. Economic modelling report [Internet]. London: NICE; 2015. Available: https://www.nice.org.uk/guidance/ng28/evidence/appendix-f-full-health-economics-report-2185320355

28. Charlton J, Rudisill C, Bhattarai N, Gulliford M. Impact of deprivation on occurrence, outcomes and health care costs of people with multiple morbidity. J Health Serv Res Policy. 2013;18: 215–223. doi:10/f5fcks

29. National Institute for Health & Care Excellence. PH25; Cardiovascular disease prevention. Costing report [Internet]. London; 2010 Jun. Available: https://www.nice.org.uk/guidance/ph25/resources/costing-report-67331053

30. National Institute for Health & Care Excellence. CG127; Hypertension in adults: diagnosis and management. Cost-effectiveness analysis [Internet]. London; 2011. Available: https://www.nice.org.uk/guidance/cg127/documents/hypertension-update-appendix-j-costeffectiveness-analysis-diagnosis2

31. Law MR, Wald NJ, Rudnicka AR. Quantifying effect of statins on low density lipoprotein cholesterol, ischaemic heart disease, and stroke: systematic review and meta-analysis. BMJ. 2003;326: 1423. doi:10/dbc3rn

32. Edwards JE, Moore RA. Statins in hypercholesterolaemia: A dose-specific meta-analysis of lipid changes in randomised, double blind trials. BMC Fam Pract. 2003;4: 18. doi:10/d8nj2c

33. Wallach-Kildemoes H, Andersen M, Diderichsen F, Lange T. Adherence to preventive statin therapy according to socioeconomic position. Eur J Clin Pharmacol. 2013;69: 1553–1563. doi:10/f248s4

34. Briggs ADM, Mytton OT, Kehlbacher A, Tiffin R, Rayner M, Scarborough P. Overall and income specific effect on prevalence of overweight and obesity of 20% sugar sweetened drink tax in UK: econometric and comparative risk assessment modelling study. BMJ. 2013;347: f6189. doi:10/gb3smc

35. Allen K, Kypridemos C, Hyseni L, Gilmore AB, Diggle P, Whitehead M, et al. The effects of maximising the UK's tobacco control score on inequalities in smoking prevalence and premature coronary heart disease mortality: a modelling study. BMC Public Health. 2016;16: 292. doi:10/f8gkd8

36. Public Health England. NHS Health Check implementation review and action plan [Internet]. London: PHE; 2013 Jul. Available: https://www.gov.uk/government/publications/nhs-health-check-implementation-review-and-action-plan

37. Hex N, Bartlett C, Wright D, Taylor M, Varley D. Estimating the current and future costs of Type 1 and Type 2 diabetes in the UK, including direct health costs and indirect societal and productivity costs. Diabet Med J Br Diabet Assoc. 2012;29: 855–862. doi:10/gc5p47

38. Liu JL, Maniadakis N, Gray A, Rayner M. The economic burden of coronary heart disease in the UK. Heart. 2002;88: 597–603. doi:10/b72f9w

39. Saka O, McGuire A, Wolfe C. Cost of stroke in the United Kingdom. Age Ageing. 2009;38: 27–32. doi:10/dhx3k5

40. Jones R, Gomes C, Lloyd K. Review of the NHS Health Check programme in Liverpool - update. Liverpool: Liverpool city council; 2016 Aug.

41. Hippisley-Cox J, Coupland C, Vinogradova Y, Robson J, Minhas R, Sheikh A, et al. Predicting cardiovascular risk in England and Wales: prospective derivation and validation of QRISK2. BMJ. 2008;336: 1475–1482. doi:10/fwp7x2

42. Collins GS, Altman DG. Predicting the 10 year risk of cardiovascular disease in the United Kingdom: independent and external validation of an updated version of QRISK2. BMJ. 2012;344: e4181. doi:10/gb3r9d

43. Bartlett S, Klerman J, Olsha L, Logan C, Blocklin M, Beauregard M, et al. Evaluation of the Healthy Incentives Pilot (HIP) Final Report [Internet]. 2014. Available: http://www.fns.usda.gov/sites/default/files/HIP-Final.pdf

44. Nnoaham KE, Sacks G, Rayner M, Mytton O, Gray A. Modelling income group differences in the health and economic impacts of targeted food taxes and subsidies. Int J Epidemiol. 2009;38: 1324–1333. doi:10/c4433b
